# Circular Network of Coregulated Sphingolipids Dictates Chronic Hypoxia Damage in Patients With Tetralogy of Fallot

**DOI:** 10.3389/fcvm.2021.780123

**Published:** 2022-01-13

**Authors:** Na Zhou, Libao Liu, Rongjun Zou, Minghui Zou, Mingxia Zhang, Fan Cao, Wenhua Liu, Huili Yuan, Guodong Huang, Li Ma, Xinxin Chen

**Affiliations:** ^1^Guangdong Provincial Key Laboratory of Research in Structural Birth Defect Disease, Heart Center, Guangzhou Women and Children's Medical Center, Guangzhou Medical University, Guangzhou, China; ^2^Guangdong Provincial Key Laboratory of Research in Structural Birth Defect Disease, Department of Extracorporeal Circulation, Guangzhou Women and Children's Medical Center, Guangzhou Medical University, Guangzhou, China; ^3^Department of Cardiothoracic Surgery, The Third Affiliated Hospital of Sun Yat-sen University, Guangzhou, China

**Keywords:** Tetralogy of Fallot, sphingolipid metabolism, metabolomic analysis, mass spectrometry analysis, cyanotic congenital heart disease

## Abstract

**Background:** Tetralogy of Fallot (TOF) is the most common cyanotic heart disease. However, the association of cardiac metabolic reprogramming changes and underlying molecular mechanisms in TOF-related chronic myocardial hypoxia damage are still unclear.

**Methods:** In this study, we combined microarray transcriptomics analysis with liquid chromatography tandem-mass spectrometry (LC–MS/MS) spectrum metabolomics analysis to establish the metabolic reprogramming that occurs in response to chronic hypoxia damage. Two Gene Expression Omnibus (GEO) datasets, GSE132176 and GSE141955, were downloaded to analyze the metabolic pathway in TOF. Then, a metabolomics analysis of the clinical samples (right atrial tissue and plasma) was performed. Additionally, an association analysis between differential metabolites and clinical phenotypes was performed. Next, four key genes related to sphingomyelin metabolism were screened and their expression was validated by real-time quantitative PCR (QT-PCR).

**Results:** The gene set enrichment analysis (GSEA) showed that sphingolipid metabolism was downregulated in TOF and the metabolomics analysis showed that multiple sphingolipids were dysregulated. Additionally, genes related to sphingomyelin metabolism were identified. We found that four core genes, UDP-Glucose Ceramide Glucosyltransferase (*UGCG*), Sphingosine-1-Phosphate Phosphatase 2 (*SGPP2*), Fatty Acid 2-Hydroxylase (*FA2H*), and Sphingosine-1-Phosphate Phosphatase 1 (*SGPP1*), were downregulated in TOF.

**Conclusion:** Sphingolipid metabolism was downregulated in TOF; however, the detailed mechanism needs further investigation.

## Introduction

Tetralogy of Fallot (TOF) is the most common cause of cyanotic congenital heart disease (CHD) and the most frequent complex CHD encountered in adulthood (1), with a prevalence of 1/3,000 births (2). TOF is considered a malformation of the cardiac outflow tract that consists of four specific postnatal structural characteristics: ventricular septal defect (VSD), anterocephalad deviation of the outflow septum with resultant overriding of the aorta, variable obstruction of the right ventricular outflow tract (RVOT) (pulmonary stenosis), and consequent hypertrophy of the right ventricle (RV) (3). The RV of patients with TOF is exposed to chronic hypoxia and hemodynamic stress (4, 5). However, the mechanism underlying the effect of a hypoxic environment on TOF pathogenesis after birth is poorly understood.

Chronic hypoxia affects cardiac metabolism and function (6), which causes metabolic reprogramming in CHD (7). Metabolomics has revealed metabolic changes in the plasma or serum of patients with CHD. Cedars et al. (8) reported that the amino acid metabolic pathway was significantly changed in adult CHD, which is consistent with the results of another study (9). Cao et al. (10) showed that uric acid and sphingolipid were increased in the serum of patients with VSD. To date, changes in the metabolomics expression profile of TOF have not been reported.

In this study, integrated bioinformatics analyses showed that sphingolipid metabolism was significantly changed in TOF. Subsequently, differential metabolomics was used to investigate the changes in the metabolites and metabolic pathways of TOF. Next, association analyses between differential sphingomyelin metabolites and important clinical phenotypes were performed. Finally, we identified four core genes involved in sphingolipid metabolism in TOF.

## Materials and Methods

### Clinical Samples

**Figure 1:**
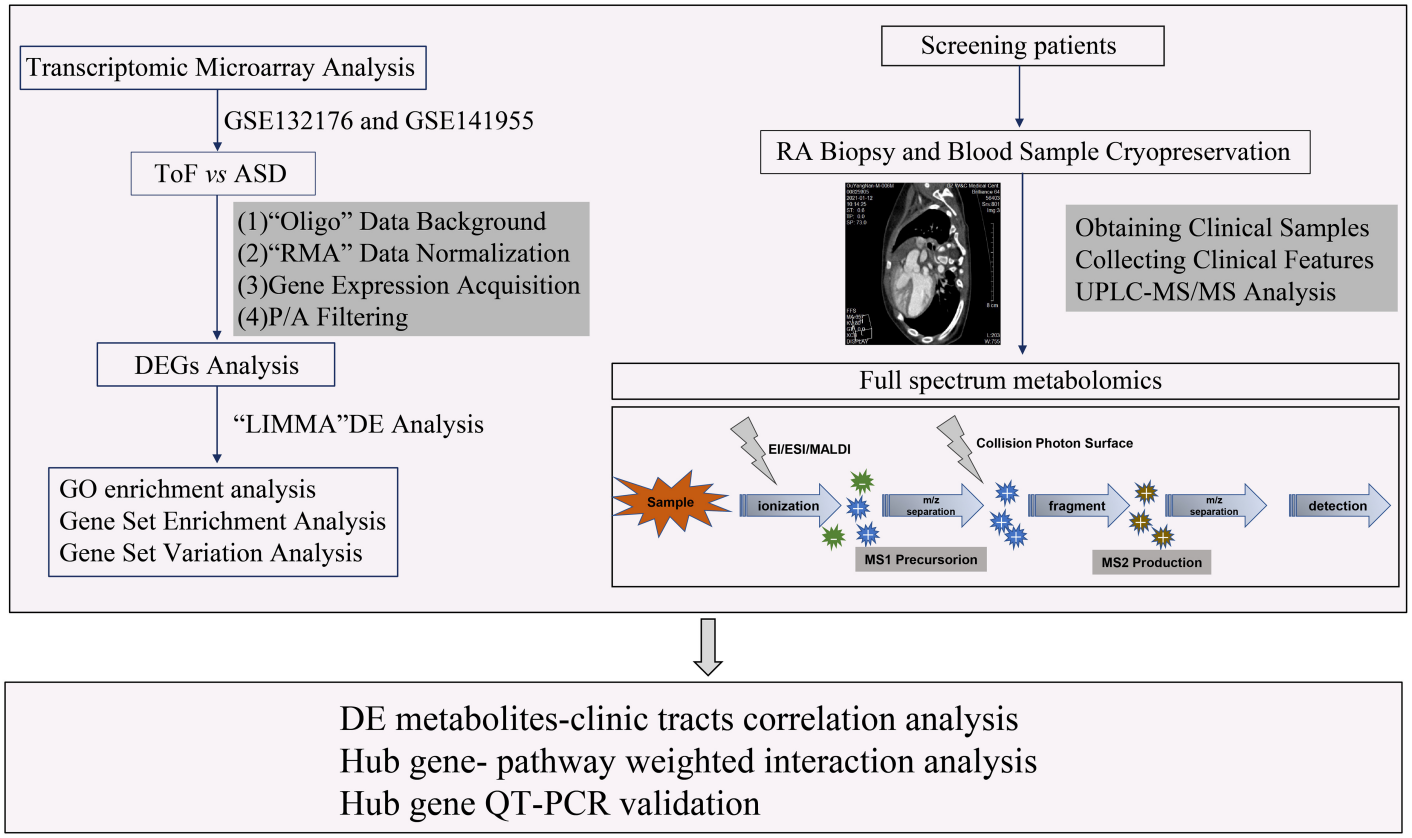
Workflow of the sphingolipid metabolism analysis of this study.

In this study, a total of 24 samples, including 12 right atrial (RA) biopsies and 12 blood sera from 6 patients with TOF and 6 patients with atrial septal defect (ASD), were collected from Guangzhou Women and Children's Medical Center between June 1, 2018 and January 1, 2020. All the research protocols for this study were approved by the Ethics Committee of the Chinese Clinical Trial Registry Center (https://www.chictr.org.cn/index.aspx; Registration number: ChiCTR-EOC-17013273) and Guangzhou Women and Children's Medical Center (approval no. from the ethics committee: 2017103101). Informed written consent was obtained from all the patients and guardians. All the patients underwent a complete physical examination. Clinical data, including medical records, ECGs, echocardiographs, and cardiac catheterization reports, were systematically reviewed (Table 1). The New York University Pediatric Heart Failure Index (NYU PHFI) and the modified Ross score were used to evaluate heart function (11). The clinical characteristics of the patients in this study are shown in Table 1. RA myocardial tissue specimens and blood sera were obtained from children affected by TOF or ASD following cardiac surgery before cardiopulmonary bypass. The harvested tissues and plasma samples were frozen in liquid nitrogen immediately after excision and stored at −80°C. The flow chart of this study shown in Figure 1.

**Table 1:** Clinical characteristics of the patients.

	**TOF**			**VSD**			***P*-value**
	**Mean**	**SD**	**IQR**	**Mean**	**SD**	**IQR**	
*N* per group	12			12			-
Age (months)	6.6	3.1	2.5, 8.0	8.3	2.3	2.0, 9.5	0.15
Weight (kg)	7.7	1.1	4.6, 7.8	6.5	1.3	1.2, 7.5	0.813
Sex (% male)	83.3	-		41.7	-		0.089
Pre-SPO_2_ (%)	84.5	7.3	76.4, 93.5	98.2	1.5	94.5–99.0	<0.001
EF	64.8	3.8	58.0, 72.0	66.7	4	60.0, 68.5	0.242
RVOT (mm)	11.2	2	9.6, 13.4	13	2.2	11.5, 14.6	0.042
RVAW (mm)	5.3	1.4	4.2, 6.5	–	–	–	–
IVS (mm)	5.3	0.6	4.6, 6.2	–	–	–	–
RVOTd (mm)	4.4	1.2	3.4, 5.1	–	–		–
McGoon	1.8	0.2	1.5, 2.1	–	–		–

*N per group, number of each group; EF, ejection fraction; RVOT, right ventricular outflow tract; RVAW, right ventricular anterior wall; IVS, interventricular septum; RVOTd, RVOT diameter*.

### Data Acquisition

High-throughput datasets GSE132176 (12) and GSE141955 (13) were downloaded from the Gene Expression Omnibus (https://www.ncbi.nlm.nih.gov/geo). GSE132176 contains RA biopsies from 10 patients with TOF and 10 patients with ASD before [precardiopulmonary bypass (CPB)] and after (post-CPB) CPB surgery. In this study, a comparative analysis was performed for the RA samples of patients with TOF and ASD before CPB surgery. The raw transcriptomic data were based on the GPL13158: Affymetrix HT HG-U133 + PM Array Plate platform, in which the sample arrays were scanned using the Affymetrix GeneChip Scanner 3000. In addition, GSE141955 contains human heart RV samples for 6 healthy donors and 7 patients with TOF. The sample gene chips were scanned by the GeneChip Scanner 3000 7G, which matched the platform of GPL17586: Affymetrix Human Transcriptome Array 2.0.

The raw CEL data of GSE132176 and GSE141955 were downloaded along with the corresponding annotation platform files. Subsequently, the “Oligo,” “robust multi-array analysis (RMA),” and “linear models for microarrays (LIMMA)” algorithms were applied to analyze the microchip raw data (14–16). The overall process was as follows: (1) perform data background processing, normalization, and gene expression acquisition; (2) filter the “non-expressed” gene/probe expression data, in which the probe with a *p*-value <0.05 was selected based on the “paCalls” method; (3) obtain the probe information and convert the expression value following the “getProbeInfo” method; and (4) perform a differentially expressed gene (DEG) analysis *via* the “LIMMA” algorithm, in which the DEGs with criteria of the Benjamini–Hochberg method corrected a *p*-value < 0.05 and log2-fold change (FC) >1.0.

### Gene Set Enrichment Analysis (GSEA) and Gene Set Variation Analysis (GSVA)

The R package “clusterProfiler” was used to perform the GSEA of hub genes with RNA sequencing (RNA-seq) data (17). In addition, the “GSVA” R package was used to find the pathways most associated with hub genes (18). *p* < 0.05 was considered as statistically significant. In this study, the maps included C5: ontology gene sets, H: hallmark gene sets, and C2: canonical pathway gene sets derived from the Kyoto Encyclopedia of Genes and Genomes (KEGG) pathway database, which were selected in this script from the Molecular Signatures Database (MSigDB) collections (http://www.gsea-msigdb.org/gsea/msigdb/collections.jsp).

### Sample Preparation and Extraction

The tissue and plasma samples were thawed on ice and metabolites were extracted with 50% methanol buffer. Briefly, 20 μl of sample was extracted with 120 μl of precooled 50% methanol, vortexed for 1 min, and incubated at room temperature for 10 min; the extraction mixture was then stored overnight at −20°C. After centrifugation at 4,000 g for 20 min, the supernatants were transferred to a new 96-well plate. The samples were stored at −80°C prior to LC-MS analysis. In addition, pooled QC samples were also prepared by combining 10 μl of each extraction mixture.

### Chromatography Analysis

The sample extracts were analyzed using an LC–electrospray ionization (ESI)–MS/MS system [ultra performance liquid and gas chromatography (UPLC)–MS/MS, ExionLC AD, https://sciex.com.cn/; MS, QTRAP® System, https://sciex.com/]. The analytical conditions were as follows: UPLC column, Thermo Accucore™ C30 (2.6 μm, 2.1 mm × 100 mm id); solvent system, A: acetonitrile/water (60/40 v/v, 0.1% formic acid, 10 mmol/l ammonium formate), B: acetonitrile/isopropanol (10/90 v/v, 0.1% formic acid, 10 mmol/l ammonium formate); gradient program, A/B (80:20 v/v) at 0 min, 70:30 v/v at 2.0 min, 40:60 v/v at 4 min, 15:85 v/v at 9 min, 10:90 v/v at 14 min, 5:95 v/v at 15.5 min, 5:95 v/v at 17.3 min, 80:20 v/v at 17.3 min, 80:20 v/v at 20 min; flow rate, 0.35 ml/min; temperature, 45°C; and injection volume: 2 μl. The effluent was alternatively connected to an ESI-triple quadrupole-linear ion trap (QTRAP)-MS.

### Mass Spectrometry Analysis

Linear ion trap (LIT) and triple quadrupole (QQQ) scans were acquired on a triple QTRAP mass spectrometer LC–MS/MS System equipped with an ESI Turbo Ion-Spray Interface, operating in positive and negative ion mode and controlled by Analyst 1.6.3 software (AB-SCIEX, Shanghai, China). The ESI source operation parameters were as follows: ion source, turbo spray; source temperature of 500°C; ion spray (IS) voltage at 5,500 V (positive) and −4,500 V(negative); ion source gas 1 (GS1), gas 2 (GS2), curtain gas (CUR) at 45, 55, and 35 psi, respectively; and collision gas collision-activated dissociation (CAD) was medium. Instrument tuning and mass calibration were performed with 10 and 100 μmol/l polypropylene glycol solutions in QQQ and LIT modes, respectively. QQQ scans were acquired as Multiple Reaction Monitoring (MRM) experiments with collision gas (nitrogen) set to 5 psi. Declustering potential (DP) and collision energy (CE) for individual MRM transitions were performed with further DP and CE optimization. A specific set of MRM transitions was monitored for each period according to the metabolites eluted within this period.

### Principal Component Analysis

Unsupervised principal component analysis (PCA) was performed by the statistics function “prcomp” within R (version 4.0.2; www.r-project.org) (19). The data were unit variance-scaled before unsupervised PCA.

### Hierarchical Cluster Analysis (HCA) and Pearson Correlation Coefficients (PCCs)

The HCA results of the samples and metabolites are presented as heatmaps with dendrograms, while PCCs between samples were calculated by the cor function in R and presented as only heatmaps (20). Both the HCA and PCC were carried out by the R package ComplexHeatmap. For the HCA, the normalized signal intensities of metabolites (unit variance scaling) were visualized as a color spectrum.

### Differential Metabolites Analysis

Significantly regulated metabolites between groups were determined by variable importance in projection (VIP) ≥ 1 and absolute log2-FC ≥ 1. VIP values were extracted from the orthogonal partial least-squares discriminant analysis (OPLS-DA) results, which also contained score plots and permutation plots and were generated using the R package MetaboAnalystR (21). The data were log2 transformed and mean centered before the OPLS-DA. To avoid overfitting, a permutation test (200 permutations) was performed.

### Kyoto Encyclopedia of Genes and Genomes Pathway Enrichment Analysis

Identified metabolites were annotated using the KEGG (http://www.kegg.jp/kegg/compound/) compound database and annotated metabolites were then mapped to the KEGG pathway database (http://www.kegg.jp/kegg/pathway.html) (22). Significantly enriched pathways were identified with a hypergeometric test *p*-value for a given list of metabolites.

### Real-time PCR (QT-PCR)

Total RNA of the tissues and plasma was obtained using TRIzol Reagent (Invitrogen, Carlsbad, California, USA) according to the protocol of the manufacturer. Next, RNA was quantified *via* the SYBR Green using the Roche Light-Cycler 480 Real-Time PCR System (Roche, Germany, UK). D-glyceraldehyde-3-phosphate dehydrogenase (GADPH) was used as the internal control. Subsequently, quantitative PCR (qPCR) was performed using a final volume of 20 μl of the SYBR Green PCR Master Mix. The primer sequences were as follows: SGPP1, F: 5'-CCATTTGTGGACCTGATTGACA-3', R: 5'- ACTTCCTAGTATCTCGGCTGTG-3'; SGPP2, 5'- TCACCGCACTCCTCATCGT-3'; UGCG, F: 5'- GAATGGCCGTCTTCGGGTT-3', R: 5'- AGGTGTAATCGGGTGTAGATGAT-3'; FA2H, F: 5'- CTGTATCTCGGCTGGTCCTACT-3', R: 5'- ATGAGGCTCCAGAGGAATGTCC-3'; and ß-actin, F: 5'- TGACGTGGACATCCGCAAAG-3', R: 5'-CTGGAAGGTGGACAGCGAGG-3'. Target values were assessed according to the 2^−Δ*ΔCT*^ method through normalization to an internal control, where the mean of the control samples was used as a calibrator.

## Results

### Identification of Sphingolipid Metabolism in TOF

**Figure 2:**
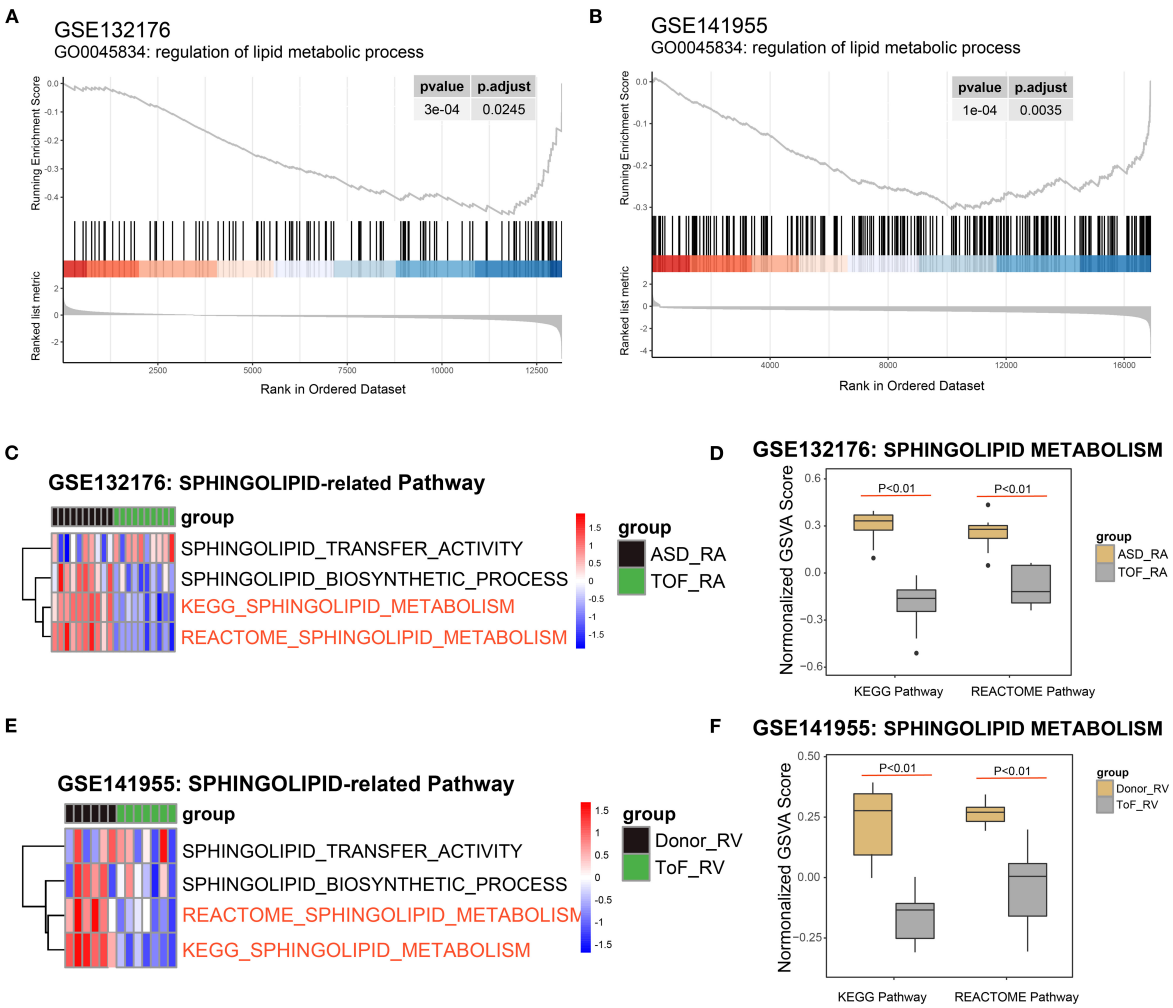
Identification of sphingolipid metabolism in Tetralogy of Fallot (TOF). **(A)** Lipid metabolic process was significantly enriched in the GSE132176 dataset by the gene set enrichment analysis (GSEA). **(B)** Lipid metabolic processes were significantly enriched in the GSE141955 dataset by the GSEA. **(C,D)** Sphingolipid metabolism was significantly downregulated in TOF compared with atrial septal defects (ASDs) by the Kyoto Encyclopedia of Genes and Genomes (KEGG) and Reactome analyses, while sphingolipid transfer activity and the sphingolipid biosynthetic process were not significantly different. **(E,F)** Sphingolipid metabolism was significantly downregulated in TOF compared with healthy donors by the KEGG and Reactome analyses, while sphingolipid transfer activity and the sphingolipid biosynthetic process were not significantly different.

Based on the powerful differential analysis performed after comparing the RA samples among the TOF and ASD data, 128 DEGs were identified, with 108 genes downregulated and 20 genes upregulated in the GSE132176 dataset. However, 627 DEGs (607 downregulated and 20 upregulated) were detected in the comparisons of RV samples among the TOF and ASD data in the GSE141955 dataset. The GSEA of the gene profile showed that 146 terms were significantly enriched in GSE132176, with 60 gene sets upregulated and 86 gene sets downregulated, while 390 terms were significantly enriched in GSE141955, with 21 gene sets upregulated and 369 gene sets downregulated (Supplementary Table 1). In addition, we found that regulation of lipid metabolic processes was significantly enriched (*p* < 0.05) and downregulated compared with ASDs or healthy donors (Figures 2A,B). Next, we focused on the pathways related to sphingolipid. Pathway analysis of GSE132176 showed that sphingolipid metabolism was significantly downregulated in the RA of TOF compared with ASD, while sphingolipid transfer activity and the sphingolipid biosynthetic process were not significantly different (Figures 2C,D). The results showed the same trend in GSE132176 (Figures 2E,F).

### Clustering Analysis of the Genes Related to Sphingolipid Metabolism and the Sphingolipid Biosynthetic Process

**Figure 3:**
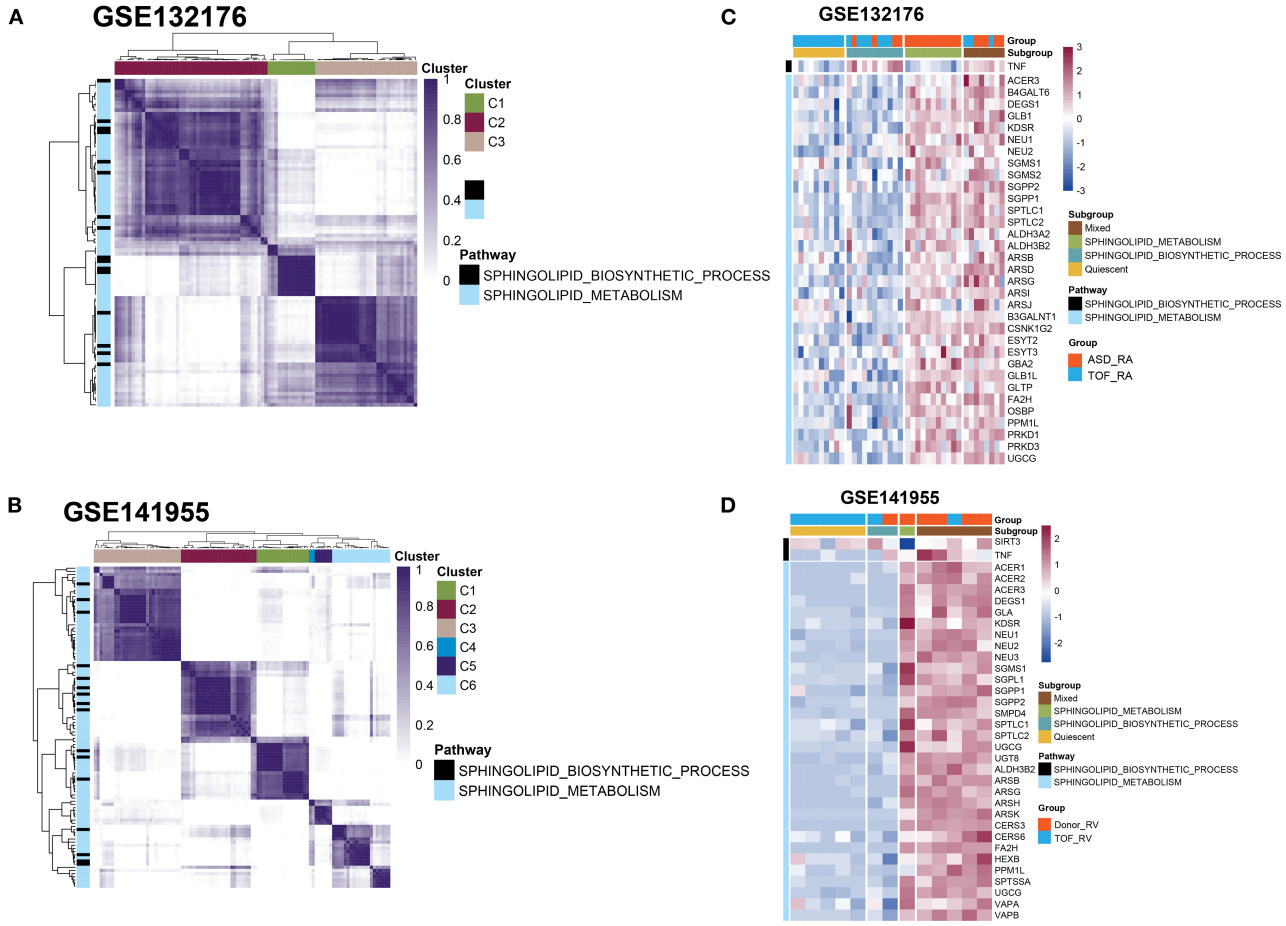
Clustering analysis of genes involved in sphingolipid metabolism and the sphingolipid biosynthetic process. **(A,C)** Heatmap showing the genes involved in sphingolipid metabolism and the sphingolipid biosynthetic process between the TOF and atrial septal defect (ASD) samples in the GSE132176 dataset. **(B,D)** Heatmap showing the genes involved in sphingolipid metabolism and the sphingolipid biosynthetic process between patients with TOF and healthy donors in the GSE141955 dataset.

Next, we performed cluster analysis on these genes involved in sphingolipid metabolism and the sphingolipid biosynthetic processes between the TOF and ASD samples in the GSE132176 dataset. We found that most genes were related to sphingolipid metabolism and only tumor necrosis factor (TNF) belonged to the sphingolipid biosynthetic process (Figure 3A and Supplementary Table 2). Additionally, in GSE141955, most genes were enriched in sphingolipid metabolism including both the *TNF* and sirtuin 3 (*SIRT3*) genes (Figure 3B and Supplementary Table 2). Moreover, the genes involved in sphingolipid metabolism and the sphingolipid biosynthetic process in GSE132176 and GSE141955 were clustered into 3 and 6 clusters, respectively (Figures 3C,D and Supplementary Table 2). Moreover, to better validate the changes in sphingolipid metabolism, we performed a metabolomics study using ASD and TOF RA biopsy and plasma samples (Figures 3C,D and Supplementary Table 2).

### Statistical Analysis of Differential Metabolites

**Figure 4:**
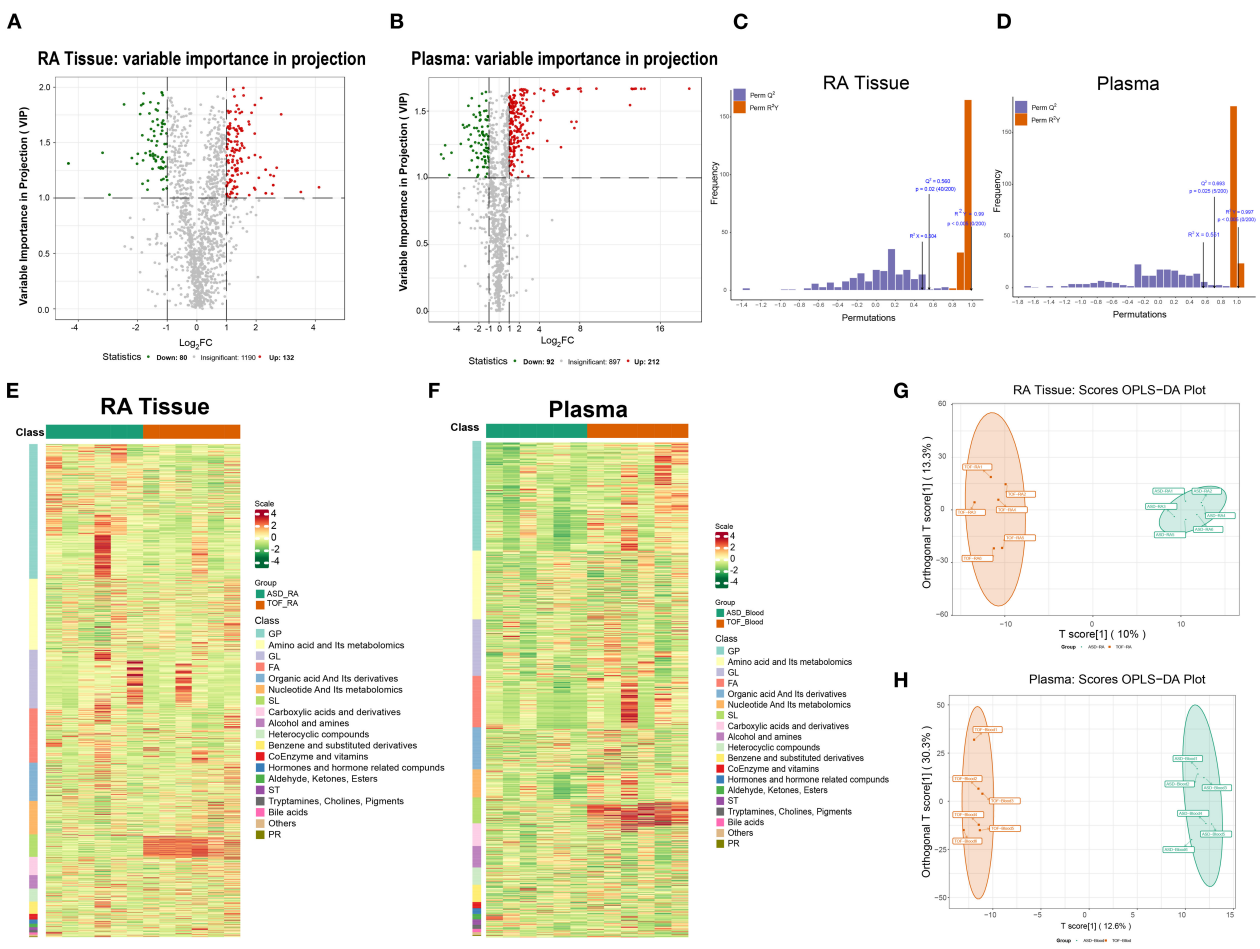
Statistical analysis of differential metabolites between the TOF and ASD samples. **(A,B)** Volcano plot showing the differential metabolites between the TOF and ASD samples. Red and green indicate upregulated and downregulated metabolites in the TOF group, respectively. **(C,D)** OPLS-DA score plot. The X-axis represents the predicted component score, while the Y-axis represents the orthogonal component score. **(E,F)** Heatmap showing the differential metabolites of the right atrium (RA) and plasma between patients with TOF and ASD. **(G,H)** Plot of the RA and plasma OPLS-DA between patients with TOF and ASD.

The differential metabolites were divided into 19 classes. To further investigate the degree of similarity and differences between the ASD and TOF groups, OPLS-DA was performed. The results identified a clear separation of the two groups in RA tissue (Figure 4A) and plasma (Figure 4B). Moreover, the OPLS-DA score plot indicated a valid model with *Q*^2^ > 0.5 (Figures 4C,D). Metabolomics profiling of the RA and plasma samples was carried out using UPLC-LC/MS. There were 212 differential metabolites in RA tissue including 132 upregulated and 80 downregulated metabolites in patients with TOF. The heatmap (Figures 4E,F) and volcano map (Figures 4G,H) showed the differential metabolites of RA and plasma between patients with TOF and ASD.

### Functional Enrichment of Differential Metabolites

**Figure 5:**
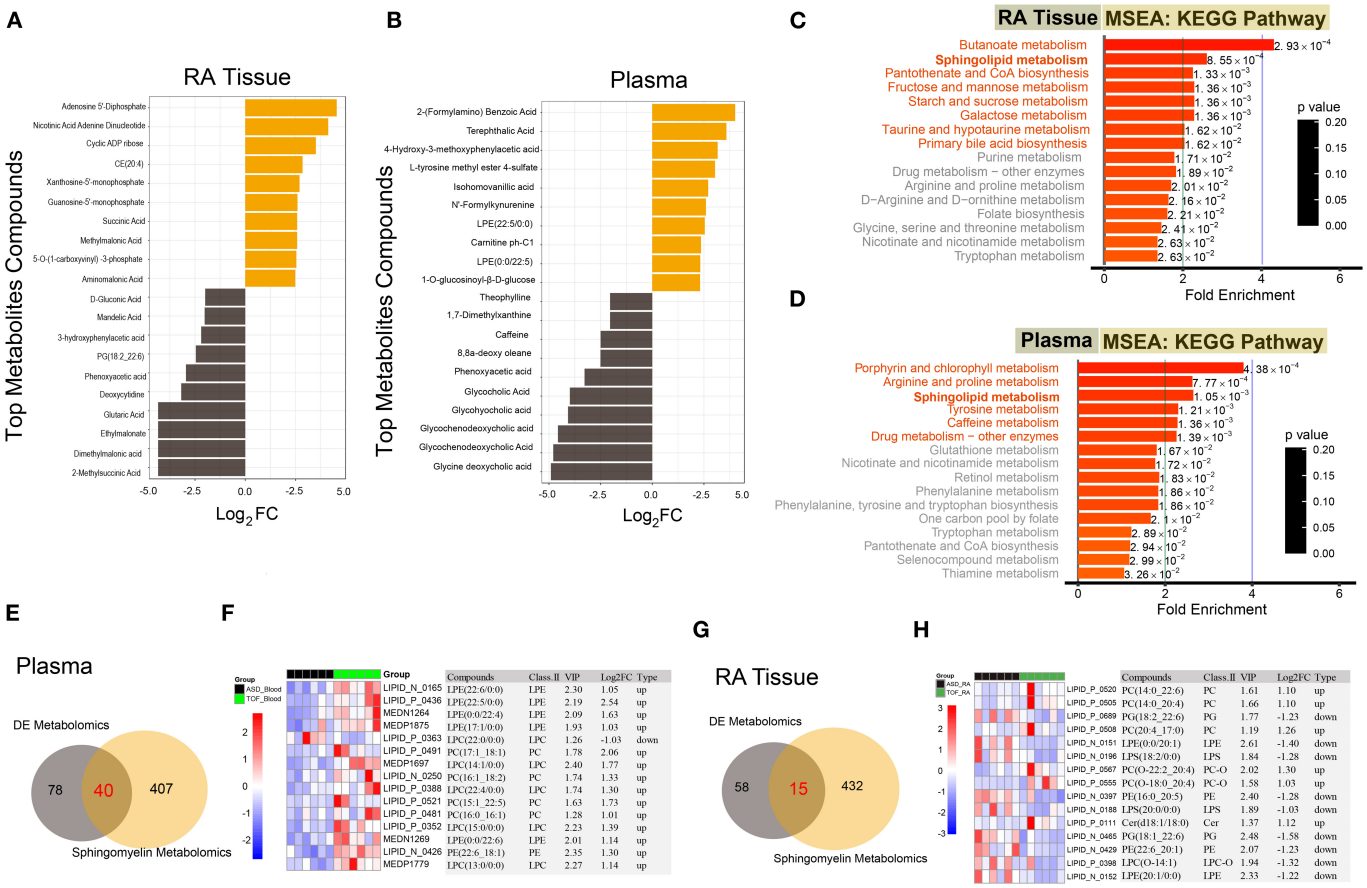
Functional enrichment of the differential metabolites and differential analysis of sphingolipid metabolism-related metabolites. **(A,B)** Top 20 differential metabolites between the TOF and ASD samples. **(C,D)** The KEGG pathway enrichment of the differential metabolites between the TOF and ASD samples. **(E)** The common differential metabolites of plasma between our metabolites and the sphingomyelin metabolite library. **(F)** Heatmap of common differential metabolites of plasma between our metabolites and the sphingomyelin metabolite library. **(G)** The common differential metabolites of RA tissue between our metabolites and the sphingomyelin metabolite library. **(H)** Heatmap of common differential metabolites of RA tissue between our metabolites and the sphingomyelin metabolite library.

Due to the multiple kinds of metabolites, we displayed the top 20 differential metabolites between the TOF and ASD samples in RA tissue and plasma (Figures 5A,B). In TOF, ten differential metabolites were upregulated and ten differential metabolites were downregulated. To understand the regulatory pathways involved in these differential metabolites, we conducted the KEGG pathway enrichment analysis. Many pathways were dysregulated. However, sphingolipid metabolism was found to be significantly enriched in RA tissues and plasma (Figures 5C,D). Next, a Venn diagram showed that there were 40 common differential metabolites of plasma between this study and the sphingomyelin metabolite library (Figure 5E and Supplementary Table 3) and the expression of the top 15 metabolites is shown in Figure 5F. Similarly, we obtained 15 differential sphingomyelin metabolites (Figure 5G and Supplementary Table 4) and detailed information on the top 15 metabolites is shown in Figure 5H.

### Association Analysis Between Differential Sphingomyelin Metabolites and Clinical Phenotypes

**Figure 6:**
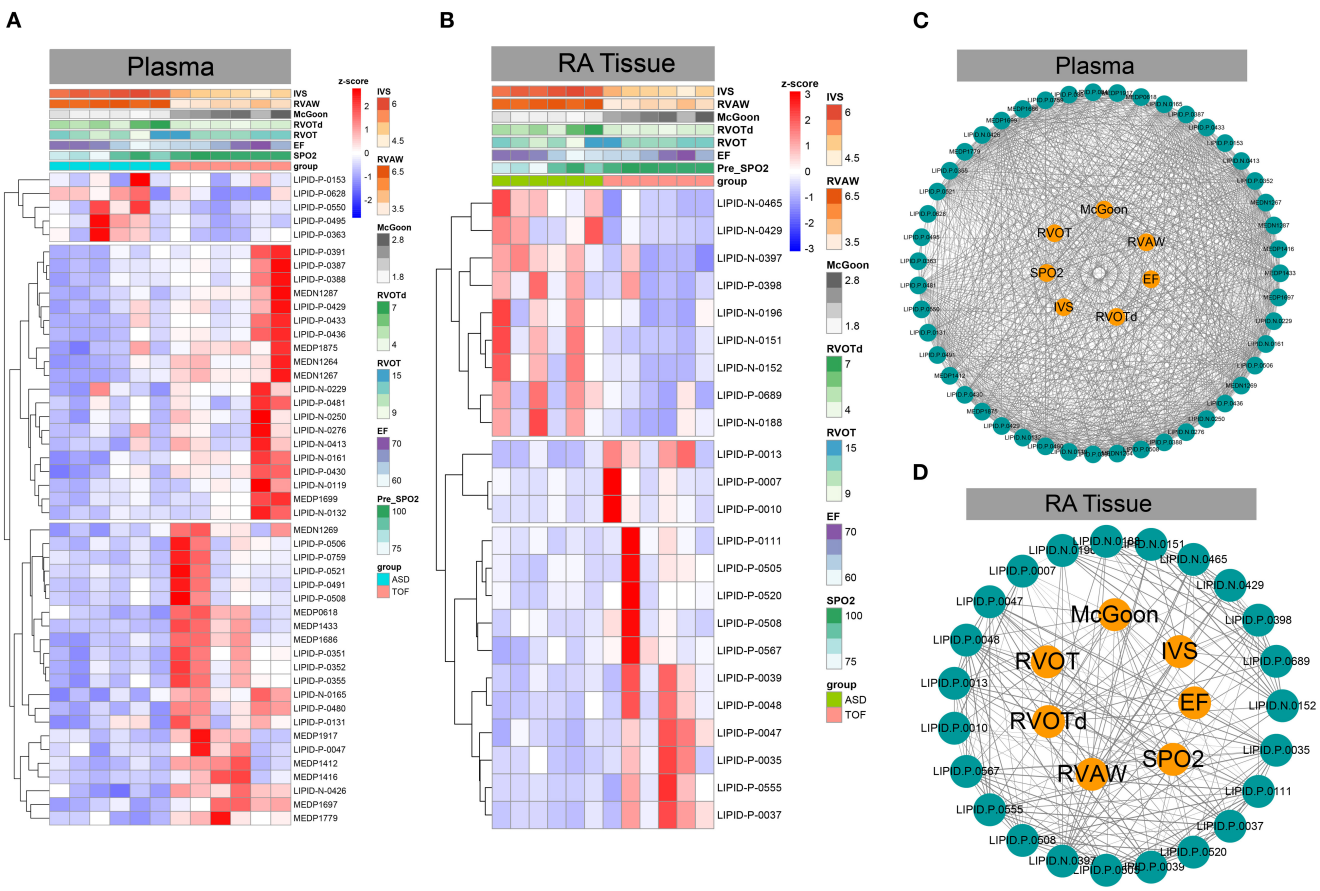
Association analysis between differential sphingomyelin metabolites and important clinical phenotypes in TOF. **(A,B)** Heatmaps based on z-scores of differential sphingomyelin metabolites and clinically associated phenotypes. **(C,D)** Correlation analysis between *z*-scores of differential sphingomyelin metabolites and clinically important phenotypes in TOF.

Subsequently, we analyzed the correlation of these differential sphingomyelin metabolites and some clinical parameters including mean transcutaneous oxygen saturation before operation (pre-SpO_2_), left ventricular ejection fraction (EF), RVOT, RVOT diameter (RVOTd), McGoon index, right ventricular anterior wall (RVAW), and ventricular septal thickness (IVS). We found that most sphingomyelin metabolites showed a positive correlation with these clinical parameters in plasma (Figures 6A–C). In this study, the metabolites LIPID.N.0132 (weighted *Pearson* = 0.306), LIPID.N.0119 (weighted *Pearson* = 0.330), and LIPID.N.0229 (weighted *Pearson* = 0.366) were significantly correlated with the EF values. The metabolites LIPID.P.0550 (weighted *Pearson* = 0.520), LIPID.P.0495 (weighted *Pearson* = 0.548), and LIPID.P.0628 (weighted *Pearson* = 0.629) were primarily correlated with IVS. The metabolites MEDN1267 (weighted *Pearson* = 0.817), LIPID.N.0426 (weighted *Pearson* = 0.842), and MEDP1697 (weighted *Pearson* = 0.882) were correlated with McGoon. The metabolites LIPID.N.0165 (weighted *Pearson* = 0.729), MEDP1875 (weighted *Pearson* = 0.741), and LIPID.P.0430 (weighted *Pearson* = 0.747) were correlated with pre-SpO_2_. The metabolites LIPID.P.0363 (weighted *Pearson* = 0.477), LIPID.P.0550 (weighted *Pearson* = 0.520), and LIPID.P.0495 (weighted *Pearson* = 0.560) were correlated with RVAW. The metabolites LIPID.P.0759 (weighted *Pearson* = 0.350), MEDN1269 (weighted *Pearson* = 0.392), and LIPID.N.0165 (weighted *Pearson* = 0.470) were correlated with RVOT. The metabolites LIPID.P.0363 (weighted *Pearson* = 0.249), LIPID.P.0153 (weighted *Pearson* = 0.398), and LIPID.P.0550 (weighted *Pearson* = 0.478) were correlated with RVOTd (Figure 6C and Supplementary Table 5).

However, some metabolites were positively correlated, while other metabolites were negatively correlated with these clinical parameters in RA tissue (Figures 6B–D). Of these metabolites, LIPID.P.0037 (weighted *Pearson* = 0.374), LIPID.P.0047 (weighted *Pearson* = 0.481), and LIPID.P.0035 (weighted *Pearson* = 0.511) were significantly correlated with the EF value. LIPID.N.0152 (weighted *Pearson* = 0.641), LIPID.N.0151 (weighted *Pearson* = 0.651), and LIPID.N.0397 (weighted *Pearson* = 0.737) were significantly correlated with IVS. LIPID.P.0555 (weighted *Pearson* = 0.570), LIPID.P.0047 (weighted *Pearson* = 0.629), and LIPID.P.0037 (weighted *Pearson* = 0.658) were correlated with McGoon. LIPID.P.0037 (weighted *Pearson* = 0.488), LIPID.P.0048 (weighted *Pearson* = 0.489), and LIPID.P.0567 (weighted *Pearson* = 0.520) were correlated with pre-SpO_2_. LIPID.N.0151 (weighted *Pearson* = 0.648), LIPID.N.0397 (weighted *Pearson* = 0.661), and LIPID.N.0465 (weighted *Pearson* = 0.742) were significantly correlated with RVAW. LIPID.P.0013 (weighted *Pearson* = 0.354), LIPID.P.0007 (weighted *Pearson* = 0.484), and LIPID.P.0010 (weighted *Pearson* = 0.500) were correlated with RVOT. Additionally, LIPID.N.0152 (weighted *Pearson* = 0.609), LIPID.N.0429 (weighted *Pearson* = 0.682), and LIPID.P.0689 (weighted *Pearson* = 0.695) were correlated with RVOTd (Figure 6D and Supplementary Table 6).

### Core Regulatory Genes Related to Sphingomyelin Metabolism in TOF

**Figure 7:**
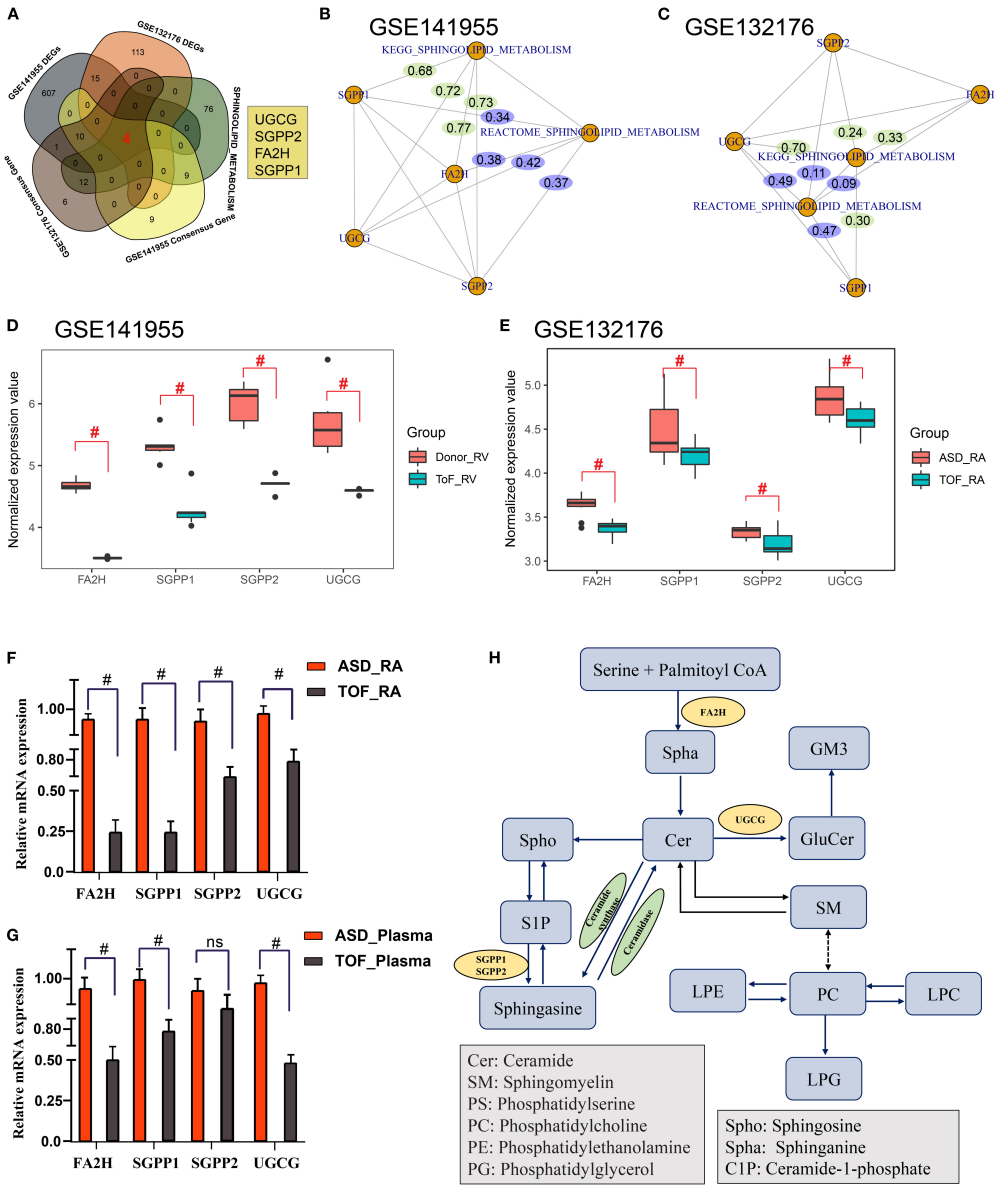
Identification of core regulatory genes related to sphingomyelin metabolism in TOF. **(A)** Common genes among the GES141955 differentially expressed genes (DEGs), GSE132176 DEGs, GES141955 consensus genes, GES132176 consensus genes, and sphingomyelin metabolism-related genes. **(B)** Gene-pathway interaction network by partial correlation analysis based on standardized gene expression values and sphingomyelin metabolic pathway scores in the GSE141955 dataset. **(C)** Gene-pathway interaction network by partial correlation analysis based on standardized gene expression values and sphingomyelin metabolic pathway scores in the GSE132176 dataset. **(D,E)** Expression of core genes in GSE132176 and GSE141955. **(F,G)** QT-PCR validation of the expression of the four core genes. **(H)** Mechanism plot of the study. ^#^*p* < 0.05.

The above metabolomics studies showed that sphingolipid metabolism was very important in TOF. By combining the analysis results for the GES141955 DEGs, GSE132176 DEGs, GES141955 consensus genes, GES132176 consensus genes, and sphingomyelin metabolism-related genes, we obtained four genes that may participate in the pathogenesis of TOF: *UGCG, SGPP2, FA2H*, and *SGPP1* (Figure 7A). Then, we constructed a gene and sphingolipid metabolism pathway network. Most gene-pathway relationships had scores >0.3 (Figures 7B,C). Additionally, GES141955 and GES132176 were significantly downregulated in the TOF RA tissue (Figures 7D,E). Moreover, QT-PCR was performed to validate the expression of the four core genes. The expression was consistent with the data in the public GEO database (Figures 7F,G). Finally, based on the KEGG pathways and our findings, we plotted the regulatory network of sphingomyelin metabolism involved in TOF (Figure 7H).

## Discussion

Dyslipidemia is an important risk factor for CHD (23). Patients with CHD with VSDs, coarctation of the aorta, and cyanosis have been proven to have lower plasma cholesterol concentration levels than patients with non-CHD (24).

Metabolic markers have an important role in regulating myocardial damage, remodeling, glycolysis, and immune/inflammatory microenvironment balance in patients with CHD (25), especially in lipid metabolism. Moreover, sphingolipid metabolism is very important in cardiovascular lipid metabolism. In the heart, sphingomyelin metabolites can alter intracellular Ca^2+^ release and ion homeostasis, which results in reduced cardiac function (26). Additionally, myocardial cells express different enzymes and receptors involved in sphingolipid metabolism and synthesize or respond to different sphingomyelin metabolites. These metabolites may mediate a variety of effects on the heart, resulting in altered cardiac function (27, 28). Furthermore, increased levels of the high-density lipoprotein sphingomyelin have been shown to be an inverse risk factor for coronary heart disease (29, 30). All of these results indicate an important role of sphingolipid in heart disease.

Tetralogy of Fallot results in chronic progressive right ventricular pressure overload and shunt hypoxemia, which leads to abnormal lipid metabolism. Oka et al. (31) found that the delay of fatty acid oxidation was harmful to heart function in newborns with cardiac hypertrophy. Xia et al. (32) reported that lipid metabolism-related proteins were decreased in TOF. In addition, proteins related to lipid metabolic processes and fatty acid oxidation in other heart diseases were also downregulated. For example, fatty acid biosynthetic process protein CYP2J2 (33), lipid transport protein clusterin and high-density lipoprotein-binding protein (34), triglyceride catabolic process protein lipoprotein lipase (35), and lipid modification protein soat2 (36). This was consistent with our finding, in which the sphingolipid metabolism pathway was downregulated. However, sphingomyelin was found to be highly expressed and identified as an independent risk factor for coronary heart disease (37, 38). These findings may indicate that the overall rate of lipid metabolic processes decreases in response to pathophysiological changes in TOF but not CHD, which decreases cardiac efficiency and energetic utilization.

Evidence has confirmed that lipid synthesis and degradation that target cardiac reactive oxygen species production and ventricular remodeling play a crucial role in maintaining cardiac function. In this study, it is important to mention the role of sphingolipid metabolism, which regulates the activity of cardiac glycolysis and structural and electrical remodeling involved in chronic hypoxic environments (39). Liu et al. (40–42) found that metabolic disorders of myocardial tissue in response to diverse pathological stimulus conditions, including ischemia reperfusion (I/R) injury, metabolic myocardial damage, and defects in hypoxia, play a critical role in the clinical prediction, diagnosis, and targeted treatment of cardiovascular disease. Wang et al. (43) demonstrated that the main cause of cardiac microvascular I/R injury is the no-reflow phenomenon after thrombolytic or revascularization therapy in patients with myocardial infarction. Their results indicate that the energy metabolism-related mitochondrial quality control (MQC) system may play an important role in the response to the pathological changes involved in oxidative stress, metabolic abnormalities, and mitochondrial dysfunction, which demonstrates a potential clinical transformation value against cardiac I/R injury (43). In addition, Wang et al. (44) demonstrated that FUN14 Domain Containing 1 (FUNDC1) exerts a significant influence on restraining myocardial stress and mitochondrial damage by targeting the mitochondrial unfolded protein response in lipopolysaccharide-induced sepsis. Li et al. (45) mapped the metabolic profile of cardiac development in pig hearts postnatally based on LC–MS/MS assays and found that early postnatal cardiac development is mainly focused on the pathway of active anabolisms of nucleotides and proteins, while a metabolic switch from glucose to fatty acids was detected in the later stage of cardiomyocyte development.

The mechanisms of sphingolipid dysregulation involved in metabolic reprogramming changes in response to chronic hypoxia damage demonstrated in this study may play an important role in glycolysis and lipid metabolism and increase the damage associated with restraining the glycolysis process, myocardium energy supply, and lipid peroxidation (25). Thus, these factors may be novel markers for the diagnosis or treatment of CHD-related chronic myocardial damage. In this study, by integrated bioinformatic analyses and metabolomics, we found that sphingolipid metabolism-related products were dysregulated in TOF. Additionally, these products showed a close correlation with clinical characteristics such as SpO_2_, EF, RVOT, RVOTd, McGoon index, RVAW, and IVS.

In another study, Xia et al. (32) also found that lipid metabolism-related proteins were associated with clinical characteristics. In addition, *FA2H, SGPP1, SGPP2*, and *UGCG*, the core sphingolipid metabolism-related genes, were downregulated in TOF. FA2H encodes fatty acid 2-hydroxylase (46), which plays a significant role in maintaining the neuronal myelin sheath (47) and influences lipid structures and metabolic signaling (48). SGPP1 and SGPP2 are two homologous sphingosine-1-phosphate (S1P) phosphatases involved in the metabolism of S1P (49). S1P has a strong effect on the development and function of the heart (50). Studies have shown that S1P regulates calcium metabolism and ionic currents in cells of the sinoatrial node, which controls heart rate (51). SGPP1 has been associated with sphingomyelins (52), which may contain genetic risk loci associated with cardiometabolic diseases (53). SGPP2 was found to play multiple roles including tumor progression (54) and inflammatory responses (55). Inflammation is very common in cardiovascular disease. However, the relationship between the inflammatory response and SGPP2 expression in TOF remains unclear. Further studies should be proposed to unveil the potential pathophysiologic implications of SGPP1 and SGPP2 in TOF. In addition, UGCG encodes glucosylceramide synthase and older Ugcg–/– mice developed severe heart failure and left ventricular dilatation and even died prematurely (56). Moreover, depletion of UGCG in the brain (57) and liver (58) causes an increase in sphingomyelin, which may confirm the relationship between UGCG and sphingomyelin metabolism in this study on TOF.

In conclusion, bioinformatics and powerful full-spectrum metabolomics techniques were used to investigate the differential metabolite profile of CHD. The results of the metabolomics analysis indicated the dysregulated level of metabolites related to sphingolipid metabolism. In addition, by bioinformatics analyses and QT-PCR validation, we obtained four sphingolipid metabolism-related genes under pathophysiological TOF conditions and this change was not detected in previous studies. This study may open new windows for understanding cardiac maladaptation in TOF. However, we still need to further elucidate the functions of these genes in TOF and the detailed regulatory relationships between these genes and differential metabolites.

However, several limitations of this study must be noted. First, RA and blood samples were selected for this study. Although TOF can be systematically analyzed from the heart to peripheral blood, it is undeniable that TOF-related myocardial damage is mainly associated with RV lesions. Limited by the difficulty and ethical requirements of obtaining RV tissue, the challenges of obtaining ample RV samples for LC–MS/MS analysis remain. Second, the abundances of different metabolites, especially sphingolipid metabolism, among the TOF and ASD groups before CPB surgery were detected. In this study, multiple metabolic pathways were identified based on differentially expressed metabolites (DEM) analysis. A weighted co-expression network analysis between clinical phenotypes and DEMs is needed. In this study, large sample and multicenter exponential investigations are warranted to validate the role of sphingolipid metabolism in cardiac remodeling in cyanotic CHD.

## Data Availability Statement

Publicly available datasets were analyzed in this study. This data can be found here: National Center for Biotechnology Information (NCBI) Gene Expression Omnibus (GEO), https://www.ncbi.nlm.nih.gov/geo/, GSE132176 and GSE141955.

## Ethics Statement

All the research protocol for this study was approved by the Ethics Committee of he Chinese Clinical Trial Registry Center (https://www.chictr.org.cn/index.aspx; Registration number: ChiCTR-EOC-17013273) and Guangzhou Women and Children's Medical Center (Approved No. of Ethics Committee: 2017103101). Written informed consent to participate in this study was provided by the participants' legal guardian/next of kin.

## Author Contributions

NZ, LL, and RZ take responsibility for all the aspects of the reliability and freedom from bias of the data presented and their discussed interpretations, they also drafted the article. MZo, MZh, FC, WL, and HY take responsibility for the statistical analyses and interpretation of the data. GH, LM, and XC take responsibility for the full-text evaluation and guidance and performed the final approval of the version to be submitted. All the authors have read and approved the final version of the manuscript.

## Funding

This study was funded by the Guangdong Peak Project (DFJH201802) and the Key Project of Natural Science Foundation of Guangdong Province (2017B030311010).

## Conflict of Interest

The authors declare that the research was conducted in the absence of any commercial or financial relationships that could be construed as a potential conflict of interest.

## Publisher's Note

All claims expressed in this article are solely those of the authors and do not necessarily represent those of their affiliated organizations, or those of the publisher, the editors and the reviewers. Any product that may be evaluated in this article, or claim that may be made by its manufacturer, is not guaranteed or endorsed by the publisher.

## Supplementary Material

Supplementary Table 1 Gene set enrichment analysis results involved the Gene Ontology terms among the GSE132176 and GSE141955 datasets.

Supplementary Table 2 Consensus cluster analysis results of the genes related to sphingolipid metabolism and the sphingolipid biosynthetic process.

Supplementary Table 3 Sphingomyelin metabolism-related differentially expressed metabolites in serum.

Supplementary Table 4 Sphingomyelin metabolism-related differentially expressed metabolites in right atrial biopsies.

Supplementary Table 5 Partial correlation between sphingomyelin-related metabolites and clinical phenotypes in patients with Tetralogy of Fallot (TOF) in serum.

Supplementary Table 6 Partial correlation between sphingomyelin-related metabolites and clinical phenotypes in patients with TOF in right atrial biopsies.
