# Supplementary material for: Circular Network of Coregulated Sphingolipids Dictates Chronic Hypoxia Damage in Patients With Tetralogy of Fallot
Source: Front Cardiovasc Med. 2022 Jan 13;8:780123. doi: 10.3389/fcvm.2021.780123 (PMC8792512; doi:10.3389/fcvm.2021.780123)
Supplement: Supplementary Table 3 — Sphingomyelin metabolism-related differentially expressed metabolites in serum. [file Table_3.pdf]

Table S3. The sphingomyelin metabolism related differentially expressed metabolites in serum.

ID	Formula	Compounds	Class I	Class II	VIP	Log2FC	Type
LIPID-P-0131	C40H79NO3	Cer(d16:1/24:0)	SL	Cer	1.71	1.04	up
MEDP1686	C20H42NO7P	LPE(15:0/0:0)	GP	LPC	2.25	1.67	up
MEDP1697	C22H44NO7P	LPC(14:1/0:0)	GP	LPC	2.40	1.77	up
MEDP1699	C24H48NO7P	LPC(0:0/16:1)	GP	LPC	1.76	1.14	up
MEDP1779	C21H44NO7P	LPC(13:0/0:0)	GP	LPC	2.27	1.14	up
LIPID-N-0119	C24H48NO7P	LPC(16:1/0:0)	GP	LPC	1.72	1.10	up
LIPID-N-0132	C28H52NO7P	LPC(20:3/0:0)	GP	LPC	1.70	1.25	up
LIPID-P-0351	C23H48NO7P	LPC(0:0/15:0)	GP	LPC	2.39	1.62	up
LIPID-P-0352	C23H48NO7P	LPC(15:0/0:0)	GP	LPC	2.23	1.39	up
LIPID-P-0355	C25H52NO7P	LPC(0:0/17:0)	GP	LPC	2.11	1.37	up
LIPID-P-0387	C30H54NO7P	LPC(0:0/22:4)	GP	LPC	1.93	1.49	up
LIPID-P-0388	C30H54NO7P	LPC(22:4/0:0)	GP	LPC	1.74	1.30	up
LIPID-P-0391	C30H52NO7P	LPC(22:5/0:0)	GP	LPC	1.84	1.42	up
LIPID-P-0363	C30H62NO7P	LPC(22:0/0:0)	GP	LPC	1.26	-1.03	down
MEDN1264	C27H48NO7P	LPE(0:0/22:4)	GP	LPE	2.09	1.63	up
MEDN1267	C27H46NO7P	LPE(0:0/22:5)	GP	LPE	2.28	2.32	up
MEDN1269	C27H44NO7P	LPE(0:0/22:6)	GP	LPE	2.01	1.14	up
MEDN1287	C21H42NO7P	LPE(0:0/16:1)	GP	LPE	2.22	1.55	up
MEDP1875	C22H44NO7P	LPE(17:1/0:0)	GP	LPE	1.93	1.03	up
LIPID-N-0161	C25H44NO7P	LPE(20:4/0:0)	GP	LPE	2.21	1.03	up
LIPID-N-0165	C27H44NO7P	LPE(22:6/0:0)	GP	LPE	2.30	1.05	up

LIPID-P-0429	C25H46NO7P	LPE(0:0/20:3)	GP	LPE	1.76	1.07	up
LIPID-P-0430	C27H50NO7P	LPE(20:3/0:0)	GP	LPE	1.94	1.31	up
LIPID-P-0433	C27H48NO7P	LPE(22:4/0:0)	GP	LPE	1.87	1.47	up
LIPID-P-0436	C27H46NO7P	LPE(22:5/0:0)	GP	LPE	2.19	2.54	up
LIPID-N-0229	C40H76NO8P	PC(14:0_18:2)	GP	PC	1.73	1.01	up
LIPID-N-0250	C42H78NO8P	PC(16:1_18:2)	GP	PC	1.74	1.33	up
LIPID-N-0276	C46H84NO8P	PC(22:4_16:0)	GP	PC	1.29	1.03	up
LIPID-P-0480	C39H76NO8P	PC(15:0_16:1)	GP	PC	2.36	1.54	up
LIPID-P-0481	C40H78NO8P	PC(16:0_16:1)	GP	PC	1.28	1.01	up
LIPID-P-0491	C43H82NO8P	PC(17:1_18:1)	GP	PC	1.78	2.06	up
LIPID-P-0506	C43H78NO8P	PC(15:0_20:4)	GP	PC	1.83	1.26	up
LIPID-P-0508	C45H80NO8P	PC(20:4_17:0)	GP	PC	1.82	1.80	up
LIPID-P-0521	C45H78NO8P	PC(15:1_22:5)	GP	PC	1.63	1.73	up
LIPID-P-0495	C48H92NO8P	PC(18:1_22:1)	GP	PC	1.75	-1.28	down
LIPID-P-0550	C44H84NO7P	PC(O-18:1_18:2)	GP	PC-O	1.48	-1.06	down
LIPID-N-0413	C43H74NO8P	PE(22:6_16:0)	GP	PE	1.72	1.08	up
LIPID-N-0426	C45H76NO8P	PE(22:6_18:1)	GP	PE	2.35	1.30	up
LIPID-P-0628	C47H80NO8P	PE(22:6_20:1)	GP	PE	1.17	-1.01	down
LIPID-P-0759	C48H95N2O6P	SM(d18:1/25:1)	SL	SM	1.91	1.86	up
