# Supplementary material for: Circular Network of Coregulated Sphingolipids Dictates Chronic Hypoxia Damage in Patients With Tetralogy of Fallot
Source: Front Cardiovasc Med. 2022 Jan 13;8:780123. doi: 10.3389/fcvm.2021.780123 (PMC8792512; doi:10.3389/fcvm.2021.780123)
Supplement: Supplementary Table 4 — Sphingomyelin metabolism-related differentially expressed metabolites in right atrial biopsies. [file Table_4.pdf]

Table S4. The sphingomyelin metabolism related differentially expressed metabolites in right atrial biopsies.

ID	Formula	Compounds	Class I	Class II	VIP	Log2FC	Type
LIPID-P-0111	C36H71NO3	Cer(d18:1/18:0)	SL	Cer	1.37	1.12	up
LIPID-P-0398	C22H46NO6P	LPC(O-14:1)	GP	LPC-O	1.94	-1.32	down
LIPID-N-0151	C25H50NO7P	LPE(0:0/20:1)	GP	LPE	2.61	-1.40	down
LIPID-N-0152	C25H50NO7P	LPE(20:1/0:0)	GP	LPE	2.33	-1.22	down
LIPID-N-0188	C26H52NO9P	LPS(20:0/0:0)	GP	LPS	1.89	-1.03	down
LIPID-N-0196	C24H44NO9P	LPS(18:2/0:0)	GP	LPS	1.84	-1.28	down
LIPID-P-0505	C42H76NO8P	PC(14:0_20:4)	GP	PC	1.66	1.10	up
LIPID-P-0508	C45H80NO8P	PC(20:4_17:0)	GP	PC	1.19	1.26	up
LIPID-P-0520	C44H76NO8P	PC(14:0_22:6)	GP	PC	1.61	1.10	up
LIPID-P-0555	C46H86NO7P	PC(O-18:0_20:4)	GP	PC-O	1.58	1.03	up
LIPID-P-0567	C50H90NO7P	PC(O-22:2_20:4)	GP	PC-O	2.02	1.30	up
LIPID-N-0397	C41H72NO8P	PE(16:0_20:5)	GP	PE	2.40	-1.28	down
LIPID-N-0429	C47H80NO8P	PE(22:6_20:1)	GP	PE	2.07	-1.23	down
LIPID-N-0465	C46H77O10P	PG(18:1_22:6)	GP	PG	2.48	-1.58	down
LIPID-P-0689	C46H75O10P	PG(18:2_22:6)	GP	PG	1.77	-1.23	down
