# Supplementary material for: Circular Network of Coregulated Sphingolipids Dictates Chronic Hypoxia Damage in Patients With Tetralogy of Fallot
Source: Front Cardiovasc Med. 2022 Jan 13;8:780123. doi: 10.3389/fcvm.2021.780123 (PMC8792512; doi:10.3389/fcvm.2021.780123)
Supplement: Supplementary Table 5 — Partial correlation between sphingomyelin-related metabolites and clinical phenotypes in patients with Tetralogy of Fallot (TOF) in serum. [file Table_5.pdf]

Table S5. The partial correlation between sphingomyelin related metabolites and clinical phenotypes in patients with TOF in serum.

source	target	pearson_corr	weight
**LIPID.N.0229**	LIPID.N.0250	0.865	0.865
**LIPID.N.0229**	LIPID.N.0276	0.744	0.744
**LIPID.N.0229**	LIPID.P.0480	0.661	0.661
**LIPID.N.0229**	LIPID.P.0481	0.830	0.830
**LIPID.N.0229**	LIPID.P.0491	0.133	0.133
**LIPID.N.0229**	LIPID.P.0506	0.346	0.346
**LIPID.N.0229**	LIPID.P.0508	0.180	0.180
**LIPID.N.0229**	LIPID.P.0521	0.194	0.194
**LIPID.N.0229**	LIPID.N.0413	0.702	0.702
**LIPID.N.0229**	LIPID.N.0426	0.310	0.310
**LIPID.N.0229**	LIPID.P.0759	0.151	0.151
**LIPID.N.0229**	MEDP1686	0.232	0.232
**LIPID.N.0229**	MEDP1697	0.452	0.452
**LIPID.N.0229**	MEDP1699	0.704	0.704
**LIPID.N.0229**	MEDP1779	0.264	0.264
**LIPID.N.0229**	LIPID.N.0119	0.781	0.781
**LIPID.N.0229**	LIPID.N.0132	0.660	0.660
**LIPID.N.0229**	LIPID.P.0351	0.341	0.341
**LIPID.N.0229**	LIPID.P.0352	0.394	0.394
**LIPID.N.0229**	LIPID.P.0355	0.361	0.361
**LIPID.N.0229**	LIPID.P.0387	0.494	0.494
**LIPID.N.0229**	LIPID.P.0388	0.549	0.549
**LIPID.N.0229**	LIPID.P.0391	0.559	0.559
**LIPID.N.0229**	MEDN1264	0.374	0.374
**LIPID.N.0229**	MEDN1267	0.334	0.334
**LIPID.N.0229**	MEDN1269	0.046	0.046
**LIPID.N.0229**	MEDN1287	0.516	0.516
**LIPID.N.0229**	MEDP1875	0.320	0.320
**LIPID.N.0229**	LIPID.N.0161	0.655	0.655
**LIPID.N.0229**	LIPID.N.0165	0.542	0.542
**LIPID.N.0229**	LIPID.P.0429	0.422	0.422
**LIPID.N.0229**	LIPID.P.0430	0.692	0.692
**LIPID.N.0229**	LIPID.P.0433	0.554	0.554
**LIPID.N.0229**	LIPID.P.0436	0.598	0.598
**LIPID.N.0229**	LIPID.P.0131	0.526	0.526
**LIPID.N.0229**	MEDP0618	0.064	0.064
**LIPID.N.0229**	MEDP1433	0.116	0.116

**LIPID.N.0229**	LIPID.P.0495	0.262	0.262
**LIPID.N.0229**	LIPID.P.0550	0.204	0.204
**LIPID.N.0229**	LIPID.P.0363	0.367	0.367
**LIPID.N.0229**	LIPID.P.0153	0.182	0.182
**LIPID.P.0480**	EF	0.011	0.011
**LIPID.P.0387**	EF	0.013	0.013
**LIPID.P.0388**	EF	0.036	0.036
**LIPID.N.0250**	LIPID.N.0276	0.926	0.926
**LIPID.N.0250**	LIPID.P.0480	0.758	0.758
**LIPID.N.0250**	LIPID.P.0481	0.851	0.851
**LIPID.N.0250**	LIPID.P.0491	0.267	0.267
**LIPID.N.0250**	LIPID.P.0506	0.431	0.431
**LIPID.N.0250**	LIPID.P.0508	0.345	0.345
**LIPID.N.0250**	LIPID.P.0521	0.305	0.305
**LIPID.N.0250**	LIPID.N.0413	0.921	0.921
**LIPID.N.0250**	LIPID.N.0426	0.417	0.417
**LIPID.N.0250**	LIPID.P.0759	0.312	0.312
**LIPID.N.0250**	MEDP1686	0.296	0.296
**LIPID.N.0250**	MEDP1697	0.351	0.351
**LIPID.N.0250**	MEDP1699	0.712	0.712
**LIPID.N.0250**	MEDP1779	0.219	0.219
**LIPID.N.0250**	LIPID.N.0119	0.847	0.847
**LIPID.N.0250**	LIPID.N.0132	0.735	0.735
**LIPID.N.0250**	LIPID.P.0351	0.385	0.385
**LIPID.N.0250**	LIPID.P.0352	0.429	0.429
**LIPID.N.0250**	LIPID.P.0355	0.450	0.450
**LIPID.N.0250**	LIPID.P.0387	0.550	0.550
**LIPID.N.0250**	LIPID.P.0388	0.598	0.598
**LIPID.N.0250**	LIPID.P.0391	0.638	0.638
**LIPID.N.0250**	MEDN1264	0.476	0.476
**LIPID.N.0250**	MEDN1267	0.402	0.402
**LIPID.N.0250**	MEDN1269	0.147	0.147
**LIPID.N.0250**	MEDN1287	0.503	0.503
**LIPID.N.0250**	MEDP1875	0.341	0.341
**LIPID.N.0250**	LIPID.N.0161	0.787	0.787
**LIPID.N.0250**	LIPID.N.0165	0.723	0.723
**LIPID.N.0250**	LIPID.P.0429	0.521	0.521
**LIPID.N.0250**	LIPID.P.0430	0.778	0.778
**LIPID.N.0250**	LIPID.P.0433	0.675	0.675
**LIPID.N.0250**	LIPID.P.0436	0.708	0.708
**LIPID.N.0250**	LIPID.P.0131	0.637	0.637
**LIPID.N.0250**	MEDP0618	0.123	0.123
**LIPID.N.0250**	MEDP1433	0.165	0.165

**LIPID.N.0250**	MEDP1917	0.009	0.009
**LIPID.N.0250**	LIPID.P.0550	0.034	0.034
**LIPID.N.0250**	LIPID.P.0363	0.053	0.053
**LIPID.N.0250**	LIPID.P.0153	0.339	0.339
**MEDN1287**	EF	0.062	0.062
**LIPID.P.0436**	EF	0.062	0.062
**MEDP1416**	EF	0.067	0.067
**LIPID.N.0161**	EF	0.084	0.084
**LIPID.N.0276**	LIPID.P.0480	0.680	0.680
**LIPID.N.0276**	LIPID.P.0481	0.858	0.858
**LIPID.N.0276**	LIPID.P.0491	0.170	0.170
**LIPID.N.0276**	LIPID.P.0506	0.314	0.314
**LIPID.N.0276**	LIPID.P.0508	0.252	0.252
**LIPID.N.0276**	LIPID.P.0521	0.180	0.180
**LIPID.N.0276**	LIPID.N.0413	0.953	0.953
**LIPID.N.0276**	LIPID.N.0426	0.310	0.310
**LIPID.N.0276**	LIPID.P.0759	0.213	0.213
**LIPID.N.0276**	MEDP1686	0.149	0.149
**LIPID.N.0276**	MEDP1697	0.163	0.163
**LIPID.N.0276**	MEDP1699	0.709	0.709
**LIPID.N.0276**	LIPID.N.0119	0.823	0.823
**LIPID.N.0276**	LIPID.N.0132	0.716	0.716
**LIPID.N.0276**	LIPID.P.0351	0.223	0.223
**LIPID.N.0276**	LIPID.P.0352	0.282	0.282
**LIPID.N.0276**	LIPID.P.0355	0.338	0.338
**LIPID.N.0276**	LIPID.P.0387	0.553	0.553
**LIPID.N.0276**	LIPID.P.0388	0.598	0.598
**LIPID.N.0276**	LIPID.P.0391	0.617	0.617
**LIPID.N.0276**	MEDN1264	0.477	0.477
**LIPID.N.0276**	MEDN1267	0.361	0.361
**LIPID.N.0276**	MEDN1269	0.029	0.029
**LIPID.N.0276**	MEDN1287	0.499	0.499
**LIPID.N.0276**	MEDP1875	0.397	0.397
**LIPID.N.0276**	LIPID.N.0161	0.711	0.711
**LIPID.N.0276**	LIPID.N.0165	0.582	0.582
**LIPID.N.0276**	LIPID.P.0429	0.590	0.590
**LIPID.N.0276**	LIPID.P.0430	0.759	0.759
**LIPID.N.0276**	LIPID.P.0433	0.753	0.753
**LIPID.N.0276**	LIPID.P.0436	0.716	0.716
**LIPID.N.0276**	LIPID.P.0131	0.578	0.578
**LIPID.N.0276**	LIPID.P.0550	0.214	0.214
**LIPID.N.0276**	LIPID.P.0363	0.066	0.066
**LIPID.N.0276**	LIPID.P.0153	0.611	0.611

**LIPID.N.0413**	EF	0.089	0.089
**LIPID.P.0391**	EF	0.106	0.106
**LIPID.N.0276**	EF	0.110	0.110
**LIPID.P.0480**	LIPID.P.0481	0.753	0.753
**LIPID.P.0480**	LIPID.P.0491	0.660	0.660
**LIPID.P.0480**	LIPID.P.0506	0.783	0.783
**LIPID.P.0480**	LIPID.P.0508	0.692	0.692
**LIPID.P.0480**	LIPID.P.0521	0.673	0.673
**LIPID.P.0480**	LIPID.N.0413	0.826	0.826
**LIPID.P.0480**	LIPID.N.0426	0.760	0.760
**LIPID.P.0480**	LIPID.P.0759	0.727	0.727
**LIPID.P.0480**	MEDP1686	0.701	0.701
**LIPID.P.0480**	MEDP1697	0.474	0.474
**LIPID.P.0480**	MEDP1699	0.617	0.617
**LIPID.P.0480**	MEDP1779	0.374	0.374
**LIPID.P.0480**	LIPID.N.0119	0.581	0.581
**LIPID.P.0480**	LIPID.N.0132	0.553	0.553
**LIPID.P.0480**	LIPID.P.0351	0.812	0.812
**LIPID.P.0480**	LIPID.P.0352	0.826	0.826
**LIPID.P.0480**	LIPID.P.0355	0.824	0.824
**LIPID.P.0480**	LIPID.P.0387	0.669	0.669
**LIPID.P.0480**	LIPID.P.0388	0.672	0.672
**LIPID.P.0480**	LIPID.P.0391	0.622	0.622
**LIPID.P.0480**	MEDN1264	0.781	0.781
**LIPID.P.0480**	MEDN1267	0.747	0.747
**LIPID.P.0480**	MEDN1269	0.674	0.674
**LIPID.P.0480**	MEDN1287	0.659	0.659
**LIPID.P.0480**	MEDP1875	0.610	0.610
**LIPID.P.0480**	LIPID.N.0161	0.741	0.741
**LIPID.P.0480**	LIPID.N.0165	0.827	0.827
**LIPID.P.0480**	LIPID.P.0429	0.706	0.706
**LIPID.P.0480**	LIPID.P.0430	0.814	0.814
**LIPID.P.0480**	LIPID.P.0433	0.704	0.704
**LIPID.P.0480**	LIPID.P.0436	0.783	0.783
**LIPID.P.0480**	LIPID.P.0131	0.835	0.835
**LIPID.P.0480**	MEDP0618	0.582	0.582
**LIPID.P.0480**	MEDP1412	0.402	0.402
**LIPID.P.0480**	MEDP1416	0.195	0.195
**LIPID.P.0480**	MEDP1433	0.623	0.623
**LIPID.P.0480**	MEDP1917	0.452	0.452
**LIPID.P.0480**	LIPID.P.0047	0.356	0.356
**LIPID.P.0480**	LIPID.P.0153	0.220	0.220
**MEDP1779**	EF	0.134	0.134

**LIPID.N.0250**	EF	0.187	0.187
**LIPID.N.0426**	EF	0.226	0.226
**MEDP1699**	EF	0.249	0.249
**LIPID.P.0481**	LIPID.P.0491	0.093	0.093
**LIPID.P.0481**	LIPID.P.0506	0.296	0.296
**LIPID.P.0481**	LIPID.P.0508	0.177	0.177
**LIPID.P.0481**	LIPID.P.0521	0.151	0.151
**LIPID.P.0481**	LIPID.N.0413	0.858	0.858
**LIPID.P.0481**	LIPID.N.0426	0.386	0.386
**LIPID.P.0481**	LIPID.P.0759	0.174	0.174
**LIPID.P.0481**	MEDP1686	0.113	0.113
**LIPID.P.0481**	MEDP1697	0.339	0.339
**LIPID.P.0481**	MEDP1699	0.904	0.904
**LIPID.P.0481**	MEDP1779	0.028	0.028
**LIPID.P.0481**	LIPID.N.0119	0.848	0.848
**LIPID.P.0481**	LIPID.N.0132	0.834	0.834
**LIPID.P.0481**	LIPID.P.0351	0.277	0.277
**LIPID.P.0481**	LIPID.P.0352	0.316	0.316
**LIPID.P.0481**	LIPID.P.0355	0.318	0.318
**LIPID.P.0481**	LIPID.P.0387	0.800	0.800
**LIPID.P.0481**	LIPID.P.0388	0.837	0.837
**LIPID.P.0481**	LIPID.P.0391	0.829	0.829
**LIPID.P.0481**	MEDN1264	0.667	0.667
**LIPID.P.0481**	MEDN1267	0.600	0.600
**LIPID.P.0481**	MEDN1269	0.151	0.151
**LIPID.P.0481**	MEDN1287	0.759	0.759
**LIPID.P.0481**	MEDP1875	0.535	0.535
**LIPID.P.0481**	LIPID.N.0161	0.793	0.793
**LIPID.P.0481**	LIPID.N.0165	0.628	0.628
**LIPID.P.0481**	LIPID.P.0429	0.765	0.765
**LIPID.P.0481**	LIPID.P.0430	0.845	0.845
**LIPID.P.0481**	LIPID.P.0433	0.870	0.870
**LIPID.P.0481**	LIPID.P.0436	0.885	0.885
**LIPID.P.0481**	LIPID.P.0131	0.518	0.518
**LIPID.P.0481**	LIPID.P.0550	0.128	0.128
**LIPID.P.0481**	LIPID.P.0363	0.163	0.163
**LIPID.P.0481**	LIPID.P.0153	0.330	0.330
**LIPID.P.0481**	EF	0.256	0.256
**MEDP1697**	EF	0.298	0.298
**LIPID.N.0132**	EF	0.306	0.306
**LIPID.N.0119**	EF	0.330	0.330
**LIPID.P.0491**	LIPID.P.0506	0.966	0.966
**LIPID.P.0491**	LIPID.P.0508	0.988	0.988

**LIPID.P.0491**	LIPID.P.0521	0.978	0.978
**LIPID.P.0491**	LIPID.N.0413	0.362	0.362
**LIPID.P.0491**	LIPID.N.0426	0.548	0.548
**LIPID.P.0491**	LIPID.P.0759	0.973	0.973
**LIPID.P.0491**	MEDP1686	0.850	0.850
**LIPID.P.0491**	MEDP1697	0.057	0.057
**LIPID.P.0491**	MEDP1779	0.285	0.285
**LIPID.P.0491**	LIPID.P.0351	0.885	0.885
**LIPID.P.0491**	LIPID.P.0352	0.920	0.920
**LIPID.P.0491**	LIPID.P.0355	0.928	0.928
**LIPID.P.0491**	LIPID.P.0387	0.034	0.034
**LIPID.P.0491**	LIPID.P.0388	0.030	0.030
**LIPID.P.0491**	MEDN1264	0.383	0.383
**LIPID.P.0491**	MEDN1267	0.336	0.336
**LIPID.P.0491**	MEDN1269	0.751	0.751
**LIPID.P.0491**	MEDN1287	0.011	0.011
**LIPID.P.0491**	MEDP1875	0.202	0.202
**LIPID.P.0491**	LIPID.N.0161	0.115	0.115
**LIPID.P.0491**	LIPID.N.0165	0.444	0.444
**LIPID.P.0491**	LIPID.P.0429	0.174	0.174
**LIPID.P.0491**	LIPID.P.0430	0.245	0.245
**LIPID.P.0491**	LIPID.P.0433	0.069	0.069
**LIPID.P.0491**	LIPID.P.0436	0.139	0.139
**LIPID.P.0491**	LIPID.P.0131	0.779	0.779
**LIPID.P.0491**	MEDP0618	0.804	0.804
**LIPID.P.0491**	MEDP1412	0.617	0.617
**LIPID.P.0491**	MEDP1416	0.233	0.233
**LIPID.P.0491**	MEDP1433	0.879	0.879
**LIPID.P.0491**	MEDP1917	0.676	0.676
**LIPID.P.0491**	LIPID.P.0047	0.533	0.533
**LIPID.P.0491**	LIPID.P.0628	0.042	0.042
**LIPID.P.0491**	LIPID.P.0153	0.156	0.156
**LIPID.N.0229**	EF	0.366	0.366
**LIPID.P.0153**	IVS	0.126	0.126
**LIPID.P.0363**	IVS	0.442	0.442
**LIPID.P.0506**	LIPID.P.0508	0.971	0.971
**LIPID.P.0506**	LIPID.P.0521	0.970	0.970
**LIPID.P.0506**	LIPID.N.0413	0.493	0.493
**LIPID.P.0506**	LIPID.N.0426	0.620	0.620
**LIPID.P.0506**	LIPID.P.0759	0.965	0.965
**LIPID.P.0506**	MEDP1686	0.832	0.832
**LIPID.P.0506**	MEDP1697	0.149	0.149
**LIPID.P.0506**	MEDP1699	0.038	0.038

**LIPID.P.0506**	MEDP1779	0.276	0.276
**LIPID.P.0506**	LIPID.N.0119	0.014	0.014
**LIPID.P.0506**	LIPID.P.0351	0.919	0.919
**LIPID.P.0506**	LIPID.P.0352	0.962	0.962
**LIPID.P.0506**	LIPID.P.0355	0.954	0.954
**LIPID.P.0506**	LIPID.P.0387	0.154	0.154
**LIPID.P.0506**	LIPID.P.0388	0.165	0.165
**LIPID.P.0506**	LIPID.P.0391	0.072	0.072
**LIPID.P.0506**	MEDN1264	0.434	0.434
**LIPID.P.0506**	MEDN1267	0.386	0.386
**LIPID.P.0506**	MEDN1269	0.707	0.707
**LIPID.P.0506**	MEDN1287	0.129	0.129
**LIPID.P.0506**	MEDP1875	0.236	0.236
**LIPID.P.0506**	LIPID.N.0161	0.244	0.244
**LIPID.P.0506**	LIPID.N.0165	0.528	0.528
**LIPID.P.0506**	LIPID.P.0429	0.257	0.257
**LIPID.P.0506**	LIPID.P.0430	0.382	0.382
**LIPID.P.0506**	LIPID.P.0433	0.186	0.186
**LIPID.P.0506**	LIPID.P.0436	0.273	0.273
**LIPID.P.0506**	LIPID.P.0131	0.851	0.851
**LIPID.P.0506**	MEDP0618	0.775	0.775
**LIPID.P.0506**	MEDP1412	0.560	0.560
**LIPID.P.0506**	MEDP1416	0.190	0.190
**LIPID.P.0506**	MEDP1433	0.854	0.854
**LIPID.P.0506**	MEDP1917	0.616	0.616
**LIPID.P.0506**	LIPID.P.0047	0.462	0.462
**LIPID.P.0506**	LIPID.P.0628	0.002	0.002
**LIPID.P.0506**	LIPID.P.0153	0.159	0.159
**LIPID.P.0550**	IVS	0.520	0.520
**LIPID.P.0495**	IVS	0.548	0.548
**LIPID.P.0628**	IVS	0.629	0.629
**LIPID.P.0508**	LIPID.P.0521	0.981	0.981
**LIPID.P.0508**	LIPID.N.0413	0.445	0.445
**LIPID.P.0508**	LIPID.N.0426	0.556	0.556
**LIPID.P.0508**	LIPID.P.0759	0.975	0.975
**LIPID.P.0508**	MEDP1686	0.801	0.801
**LIPID.P.0508**	MEDP1697	0.035	0.035
**LIPID.P.0508**	MEDP1779	0.244	0.244
**LIPID.P.0508**	LIPID.P.0351	0.862	0.862
**LIPID.P.0508**	LIPID.P.0352	0.904	0.904
**LIPID.P.0508**	LIPID.P.0355	0.914	0.914
**LIPID.P.0508**	LIPID.P.0387	0.081	0.081
**LIPID.P.0508**	LIPID.P.0388	0.086	0.086

**LIPID.P.0508**	MEDN1264	0.404	0.404
**LIPID.P.0508**	MEDN1267	0.345	0.345
**LIPID.P.0508**	MEDN1269	0.713	0.713
**LIPID.P.0508**	MEDN1287	0.041	0.041
**LIPID.P.0508**	MEDP1875	0.191	0.191
**LIPID.P.0508**	LIPID.N.0161	0.162	0.162
**LIPID.P.0508**	LIPID.N.0165	0.472	0.472
**LIPID.P.0508**	LIPID.P.0429	0.215	0.215
**LIPID.P.0508**	LIPID.P.0430	0.291	0.291
**LIPID.P.0508**	LIPID.P.0433	0.130	0.130
**LIPID.P.0508**	LIPID.P.0436	0.201	0.201
**LIPID.P.0508**	LIPID.P.0131	0.796	0.796
**LIPID.P.0508**	MEDP0618	0.757	0.757
**LIPID.P.0508**	MEDP1412	0.551	0.551
**LIPID.P.0508**	MEDP1416	0.147	0.147
**LIPID.P.0508**	MEDP1433	0.833	0.833
**LIPID.P.0508**	MEDP1917	0.588	0.588
**LIPID.P.0508**	LIPID.P.0047	0.429	0.429
**LIPID.P.0508**	LIPID.P.0628	0.067	0.067
**LIPID.P.0508**	LIPID.P.0153	0.186	0.186
**RVOTd**	IVS	0.678	0.678
**RVAW**	IVS	0.877	0.877
**LIPID.N.0276**	McGoon	0.124	0.124
**LIPID.P.0521**	LIPID.N.0413	0.380	0.380
**LIPID.P.0521**	LIPID.N.0426	0.507	0.507
**LIPID.P.0521**	LIPID.P.0759	0.977	0.977
**LIPID.P.0521**	MEDP1686	0.771	0.771
**LIPID.P.0521**	MEDP1697	0.008	0.008
**LIPID.P.0521**	MEDP1779	0.273	0.273
**LIPID.P.0521**	LIPID.P.0351	0.851	0.851
**LIPID.P.0521**	LIPID.P.0352	0.897	0.897
**LIPID.P.0521**	LIPID.P.0355	0.884	0.884
**LIPID.P.0521**	LIPID.P.0387	0.052	0.052
**LIPID.P.0521**	LIPID.P.0388	0.056	0.056
**LIPID.P.0521**	MEDN1264	0.365	0.365
**LIPID.P.0521**	MEDN1267	0.319	0.319
**LIPID.P.0521**	MEDN1269	0.723	0.723
**LIPID.P.0521**	MEDN1287	0.010	0.010
**LIPID.P.0521**	MEDP1875	0.115	0.115
**LIPID.P.0521**	LIPID.N.0161	0.118	0.118
**LIPID.P.0521**	LIPID.N.0165	0.460	0.460
**LIPID.P.0521**	LIPID.P.0429	0.164	0.164
**LIPID.P.0521**	LIPID.P.0430	0.225	0.225

**LIPID.P.0521**	LIPID.P.0433	0.060	0.060
**LIPID.P.0521**	LIPID.P.0436	0.153	0.153
**LIPID.P.0521**	LIPID.P.0131	0.753	0.753
**LIPID.P.0521**	MEDP0618	0.774	0.774
**LIPID.P.0521**	MEDP1412	0.514	0.514
**LIPID.P.0521**	MEDP1416	0.119	0.119
**LIPID.P.0521**	MEDP1433	0.837	0.837
**LIPID.P.0521**	MEDP1917	0.636	0.636
**LIPID.P.0521**	LIPID.P.0047	0.451	0.451
**LIPID.P.0521**	LIPID.P.0628	0.078	0.078
**LIPID.P.0521**	LIPID.P.0153	0.068	0.068
**EF**	McGoon	0.133	0.133
**RVOT**	McGoon	0.258	0.258
**LIPID.N.0250**	McGoon	0.282	0.282
**LIPID.N.0413**	LIPID.N.0426	0.501	0.501
**LIPID.N.0413**	LIPID.P.0759	0.431	0.431
**LIPID.N.0413**	MEDP1686	0.331	0.331
**LIPID.N.0413**	MEDP1697	0.250	0.250
**LIPID.N.0413**	MEDP1699	0.710	0.710
**LIPID.N.0413**	MEDP1779	0.129	0.129
**LIPID.N.0413**	LIPID.N.0119	0.776	0.776
**LIPID.N.0413**	LIPID.N.0132	0.715	0.715
**LIPID.N.0413**	LIPID.P.0351	0.423	0.423
**LIPID.N.0413**	LIPID.P.0352	0.467	0.467
**LIPID.N.0413**	LIPID.P.0355	0.511	0.511
**LIPID.N.0413**	LIPID.P.0387	0.633	0.633
**LIPID.N.0413**	LIPID.P.0388	0.664	0.664
**LIPID.N.0413**	LIPID.P.0391	0.666	0.666
**LIPID.N.0413**	MEDN1264	0.619	0.619
**LIPID.N.0413**	MEDN1267	0.529	0.529
**LIPID.N.0413**	MEDN1269	0.273	0.273
**LIPID.N.0413**	MEDN1287	0.566	0.566
**LIPID.N.0413**	MEDP1875	0.451	0.451
**LIPID.N.0413**	LIPID.N.0161	0.770	0.770
**LIPID.N.0413**	LIPID.N.0165	0.736	0.736
**LIPID.N.0413**	LIPID.P.0429	0.667	0.667
**LIPID.N.0413**	LIPID.P.0430	0.800	0.800
**LIPID.N.0413**	LIPID.P.0433	0.784	0.784
**LIPID.N.0413**	LIPID.P.0436	0.796	0.796
**LIPID.N.0413**	LIPID.P.0131	0.663	0.663
**LIPID.N.0413**	MEDP0618	0.175	0.175
**LIPID.N.0413**	MEDP1412	0.012	0.012
**LIPID.N.0413**	MEDP1433	0.204	0.204

**LIPID.N.0413**	MEDP1917	0.051	0.051
**LIPID.N.0413**	LIPID.P.0153	0.486	0.486
**LIPID.P.0521**	McGoon	0.284	0.284
**LIPID.P.0508**	McGoon	0.301	0.301
**LIPID.N.0229**	McGoon	0.307	0.307
**LIPID.N.0413**	McGoon	0.309	0.309
**LIPID.N.0426**	LIPID.P.0759	0.638	0.638
**LIPID.N.0426**	MEDP1686	0.760	0.760
**LIPID.N.0426**	MEDP1697	0.674	0.674
**LIPID.N.0426**	MEDP1699	0.386	0.386
**LIPID.N.0426**	MEDP1779	0.576	0.576
**LIPID.N.0426**	LIPID.N.0119	0.352	0.352
**LIPID.N.0426**	LIPID.N.0132	0.357	0.357
**LIPID.N.0426**	LIPID.P.0351	0.809	0.809
**LIPID.N.0426**	LIPID.P.0352	0.736	0.736
**LIPID.N.0426**	LIPID.P.0355	0.734	0.734
**LIPID.N.0426**	LIPID.P.0387	0.415	0.415
**LIPID.N.0426**	LIPID.P.0388	0.378	0.378
**LIPID.N.0426**	LIPID.P.0391	0.360	0.360
**LIPID.N.0426**	MEDN1264	0.545	0.545
**LIPID.N.0426**	MEDN1267	0.619	0.619
**LIPID.N.0426**	MEDN1269	0.599	0.599
**LIPID.N.0426**	MEDN1287	0.496	0.496
**LIPID.N.0426**	MEDP1875	0.517	0.517
**LIPID.N.0426**	LIPID.N.0161	0.509	0.509
**LIPID.N.0426**	LIPID.N.0165	0.595	0.595
**LIPID.N.0426**	LIPID.P.0429	0.407	0.407
**LIPID.N.0426**	LIPID.P.0430	0.527	0.527
**LIPID.N.0426**	LIPID.P.0433	0.421	0.421
**LIPID.N.0426**	LIPID.P.0436	0.534	0.534
**LIPID.N.0426**	LIPID.P.0131	0.520	0.520
**LIPID.N.0426**	MEDP0618	0.777	0.777
**LIPID.N.0426**	MEDP1412	0.777	0.777
**LIPID.N.0426**	MEDP1416	0.646	0.646
**LIPID.N.0426**	MEDP1433	0.733	0.733
**LIPID.N.0426**	MEDP1917	0.581	0.581
**LIPID.N.0426**	LIPID.P.0047	0.568	0.568
**LIPID.N.0426**	LIPID.P.0153	0.015	0.015
**LIPID.P.0491**	McGoon	0.320	0.320
**LIPID.P.0131**	McGoon	0.363	0.363
**LIPID.P.0481**	McGoon	0.363	0.363
**LIPID.P.0506**	McGoon	0.379	0.379
**LIPID.P.0759**	MEDP1686	0.815	0.815

**LIPID.P.0759**	MEDP1697	0.082	0.082
**LIPID.P.0759**	MEDP1779	0.303	0.303
**LIPID.P.0759**	LIPID.P.0351	0.889	0.889
**LIPID.P.0759**	LIPID.P.0352	0.913	0.913
**LIPID.P.0759**	LIPID.P.0355	0.910	0.910
**LIPID.P.0759**	LIPID.P.0387	0.119	0.119
**LIPID.P.0759**	LIPID.P.0388	0.109	0.109
**LIPID.P.0759**	LIPID.P.0391	0.024	0.024
**LIPID.P.0759**	MEDN1264	0.428	0.428
**LIPID.P.0759**	MEDN1267	0.397	0.397
**LIPID.P.0759**	MEDN1269	0.767	0.767
**LIPID.P.0759**	MEDN1287	0.084	0.084
**LIPID.P.0759**	MEDP1875	0.197	0.197
**LIPID.P.0759**	LIPID.N.0161	0.185	0.185
**LIPID.P.0759**	LIPID.N.0165	0.515	0.515
**LIPID.P.0759**	LIPID.P.0429	0.231	0.231
**LIPID.P.0759**	LIPID.P.0430	0.280	0.280
**LIPID.P.0759**	LIPID.P.0433	0.134	0.134
**LIPID.P.0759**	LIPID.P.0436	0.230	0.230
**LIPID.P.0759**	LIPID.P.0131	0.756	0.756
**LIPID.P.0759**	MEDP0618	0.834	0.834
**LIPID.P.0759**	MEDP1412	0.603	0.603
**LIPID.P.0759**	MEDP1416	0.217	0.217
**LIPID.P.0759**	MEDP1433	0.873	0.873
**LIPID.P.0759**	MEDP1917	0.703	0.703
**LIPID.P.0759**	LIPID.P.0047	0.558	0.558
**LIPID.P.0759**	LIPID.P.0628	0.027	0.027
**LIPID.P.0759**	LIPID.P.0153	0.096	0.096
**LIPID.P.0759**	McGoon	0.386	0.386
**LIPID.N.0119**	McGoon	0.413	0.413
**LIPID.N.0132**	McGoon	0.490	0.490
**MEDP1686**	MEDP1697	0.518	0.518
**MEDP1686**	MEDP1699	0.053	0.053
**MEDP1686**	MEDP1779	0.600	0.600
**MEDP1686**	LIPID.N.0119	0.067	0.067
**MEDP1686**	LIPID.N.0132	0.017	0.017
**MEDP1686**	LIPID.P.0351	0.957	0.957
**MEDP1686**	LIPID.P.0352	0.931	0.931
**MEDP1686**	LIPID.P.0355	0.938	0.938
**MEDP1686**	LIPID.P.0387	0.176	0.176
**MEDP1686**	LIPID.P.0388	0.146	0.146
**MEDP1686**	LIPID.P.0391	0.086	0.086
**MEDP1686**	MEDN1264	0.482	0.482

**MEDP1686**	MEDN1267	0.510	0.510
**MEDP1686**	MEDN1269	0.805	0.805
**MEDP1686**	MEDN1287	0.241	0.241
**MEDP1686**	MEDP1875	0.458	0.458
**MEDP1686**	LIPID.N.0161	0.329	0.329
**MEDP1686**	LIPID.N.0165	0.569	0.569
**MEDP1686**	LIPID.P.0429	0.256	0.256
**MEDP1686**	LIPID.P.0430	0.408	0.408
**MEDP1686**	LIPID.P.0433	0.180	0.180
**MEDP1686**	LIPID.P.0436	0.264	0.264
**MEDP1686**	LIPID.P.0131	0.722	0.722
**MEDP1686**	MEDP0618	0.889	0.889
**MEDP1686**	MEDP1412	0.870	0.870
**MEDP1686**	MEDP1416	0.665	0.665
**MEDP1686**	MEDP1433	0.953	0.953
**MEDP1686**	MEDP1917	0.798	0.798
**MEDP1686**	LIPID.P.0047	0.735	0.735
**MEDP1686**	LIPID.P.0153	0.044	0.044
**MEDP1917**	McGoon	0.505	0.505
**LIPID.P.0433**	McGoon	0.513	0.513
**LIPID.P.0047**	McGoon	0.516	0.516
**MEDP1697**	MEDP1699	0.570	0.570
**MEDP1697**	MEDP1779	0.719	0.719
**MEDP1697**	LIPID.N.0119	0.561	0.561
**MEDP1697**	LIPID.N.0132	0.590	0.590
**MEDP1697**	LIPID.P.0351	0.484	0.484
**MEDP1697**	LIPID.P.0352	0.386	0.386
**MEDP1697**	LIPID.P.0355	0.351	0.351
**MEDP1697**	LIPID.P.0387	0.523	0.523
**MEDP1697**	LIPID.P.0388	0.496	0.496
**MEDP1697**	LIPID.P.0391	0.519	0.519
**MEDP1697**	MEDN1264	0.470	0.470
**MEDP1697**	MEDN1267	0.593	0.593
**MEDP1697**	MEDN1269	0.397	0.397
**MEDP1697**	MEDN1287	0.647	0.647
**MEDP1697**	MEDP1875	0.588	0.588
**MEDP1697**	LIPID.N.0161	0.656	0.656
**MEDP1697**	LIPID.N.0165	0.568	0.568
**MEDP1697**	LIPID.P.0429	0.403	0.403
**MEDP1697**	LIPID.P.0430	0.598	0.598
**MEDP1697**	LIPID.P.0433	0.421	0.421
**MEDP1697**	LIPID.P.0436	0.518	0.518
**MEDP1697**	LIPID.P.0131	0.264	0.264

**MEDP1697**	MEDP0618	0.403	0.403
**MEDP1697**	MEDP1412	0.581	0.581
**MEDP1697**	MEDP1416	0.755	0.755
**MEDP1697**	MEDP1433	0.428	0.428
**MEDP1697**	MEDP1917	0.323	0.323
**MEDP1697**	LIPID.P.0047	0.364	0.364
**MEDP1699**	McGoon	0.540	0.540
**LIPID.P.0355**	McGoon	0.549	0.549
**LIPID.P.0391**	McGoon	0.550	0.550
**LIPID.P.0429**	McGoon	0.566	0.566
**MEDP1699**	MEDP1779	0.177	0.177
**MEDP1699**	LIPID.N.0119	0.906	0.906
**MEDP1699**	LIPID.N.0132	0.954	0.954
**MEDP1699**	LIPID.P.0351	0.156	0.156
**MEDP1699**	LIPID.P.0352	0.142	0.142
**MEDP1699**	LIPID.P.0355	0.143	0.143
**MEDP1699**	LIPID.P.0387	0.913	0.913
**MEDP1699**	LIPID.P.0388	0.925	0.925
**MEDP1699**	LIPID.P.0391	0.937	0.937
**MEDP1699**	MEDN1264	0.732	0.732
**MEDP1699**	MEDN1267	0.717	0.717
**MEDP1699**	MEDN1269	0.165	0.165
**MEDP1699**	MEDN1287	0.915	0.915
**MEDP1699**	MEDP1875	0.693	0.693
**MEDP1699**	LIPID.N.0161	0.883	0.883
**MEDP1699**	LIPID.N.0165	0.632	0.632
**MEDP1699**	LIPID.P.0429	0.841	0.841
**MEDP1699**	LIPID.P.0430	0.879	0.879
**MEDP1699**	LIPID.P.0433	0.927	0.927
**MEDP1699**	LIPID.P.0436	0.936	0.936
**MEDP1699**	LIPID.P.0131	0.331	0.331
**MEDP1699**	LIPID.P.0153	0.160	0.160
**LIPID.P.0388**	McGoon	0.570	0.570
**LIPID.P.0352**	McGoon	0.582	0.582
**LIPID.P.0430**	McGoon	0.600	0.600
**MEDP1433**	McGoon	0.613	0.613
**MEDP1779**	LIPID.N.0119	0.232	0.232
**MEDP1779**	LIPID.N.0132	0.182	0.182
**MEDP1779**	LIPID.P.0351	0.516	0.516
**MEDP1779**	LIPID.P.0352	0.434	0.434
**MEDP1779**	LIPID.P.0355	0.389	0.389
**MEDP1779**	LIPID.P.0387	0.156	0.156
**MEDP1779**	LIPID.P.0388	0.097	0.097

**MEDP1779**	LIPID.P.0391	0.129	0.129
**MEDP1779**	MEDN1264	0.292	0.292
**MEDP1779**	MEDN1267	0.419	0.419
**MEDP1779**	MEDN1269	0.477	0.477
**MEDP1779**	MEDN1287	0.316	0.316
**MEDP1779**	MEDP1875	0.319	0.319
**MEDP1779**	LIPID.N.0161	0.339	0.339
**MEDP1779**	LIPID.N.0165	0.451	0.451
**MEDP1779**	LIPID.P.0429	0.093	0.093
**MEDP1779**	LIPID.P.0430	0.205	0.205
**MEDP1779**	LIPID.P.0433	0.090	0.090
**MEDP1779**	LIPID.P.0436	0.205	0.205
**MEDP1779**	LIPID.P.0131	0.123	0.123
**MEDP1779**	MEDP0618	0.672	0.672
**MEDP1779**	MEDP1412	0.667	0.667
**MEDP1779**	MEDP1416	0.736	0.736
**MEDP1779**	MEDP1433	0.593	0.593
**MEDP1779**	MEDP1917	0.599	0.599
**MEDP1779**	LIPID.P.0047	0.477	0.477
**LIPID.P.0387**	McGoon	0.625	0.625
**LIPID.P.0436**	McGoon	0.627	0.627
**MEDP0618**	McGoon	0.628	0.628
**LIPID.N.0161**	McGoon	0.629	0.629
**LIPID.N.0119**	LIPID.N.0132	0.951	0.951
**LIPID.N.0119**	LIPID.P.0351	0.116	0.116
**LIPID.N.0119**	LIPID.P.0352	0.108	0.108
**LIPID.N.0119**	LIPID.P.0355	0.125	0.125
**LIPID.N.0119**	LIPID.P.0387	0.740	0.740
**LIPID.N.0119**	LIPID.P.0388	0.765	0.765
**LIPID.N.0119**	LIPID.P.0391	0.834	0.834
**LIPID.N.0119**	MEDN1264	0.529	0.529
**LIPID.N.0119**	MEDN1267	0.509	0.509
**LIPID.N.0119**	MEDN1269	0.024	0.024
**LIPID.N.0119**	MEDN1287	0.733	0.733
**LIPID.N.0119**	MEDP1875	0.515	0.515
**LIPID.N.0119**	LIPID.N.0161	0.897	0.897
**LIPID.N.0119**	LIPID.N.0165	0.671	0.671
**LIPID.N.0119**	LIPID.P.0429	0.649	0.649
**LIPID.N.0119**	LIPID.P.0430	0.830	0.830
**LIPID.N.0119**	LIPID.P.0433	0.805	0.805
**LIPID.N.0119**	LIPID.P.0436	0.817	0.817
**LIPID.N.0119**	LIPID.P.0131	0.341	0.341
**LIPID.N.0119**	MEDP1416	0.015	0.015

**LIPID.N.0119**	LIPID.P.0153	0.212	0.212
**LIPID.N.0165**	McGoon	0.643	0.643
**LIPID.P.0480**	McGoon	0.651	0.651
**Pre_SPO2**	McGoon	0.654	0.654
**MEDP1686**	McGoon	0.665	0.665
**LIPID.N.0132**	LIPID.P.0351	0.090	0.090
**LIPID.N.0132**	LIPID.P.0352	0.065	0.065
**LIPID.N.0132**	LIPID.P.0355	0.074	0.074
**LIPID.N.0132**	LIPID.P.0387	0.865	0.865
**LIPID.N.0132**	LIPID.P.0388	0.884	0.884
**LIPID.N.0132**	LIPID.P.0391	0.935	0.935
**LIPID.N.0132**	MEDN1264	0.628	0.628
**LIPID.N.0132**	MEDN1267	0.626	0.626
**LIPID.N.0132**	MEDN1269	0.115	0.115
**LIPID.N.0132**	MEDN1287	0.823	0.823
**LIPID.N.0132**	MEDP1875	0.562	0.562
**LIPID.N.0132**	LIPID.N.0161	0.928	0.928
**LIPID.N.0132**	LIPID.N.0165	0.702	0.702
**LIPID.N.0132**	LIPID.P.0429	0.754	0.754
**LIPID.N.0132**	LIPID.P.0430	0.854	0.854
**LIPID.N.0132**	LIPID.P.0433	0.859	0.859
**LIPID.N.0132**	LIPID.P.0436	0.887	0.887
**LIPID.N.0132**	LIPID.P.0131	0.289	0.289
**LIPID.N.0132**	LIPID.P.0153	0.069	0.069
**LIPID.P.0351**	McGoon	0.682	0.682
**MEDN1269**	McGoon	0.690	0.690
**MEDP1412**	McGoon	0.696	0.696
**MEDN1264**	McGoon	0.702	0.702
**LIPID.P.0351**	LIPID.P.0352	0.987	0.987
**LIPID.P.0351**	LIPID.P.0355	0.974	0.974
**LIPID.P.0351**	LIPID.P.0387	0.281	0.281
**LIPID.P.0351**	LIPID.P.0388	0.265	0.265
**LIPID.P.0351**	LIPID.P.0391	0.191	0.191
**LIPID.P.0351**	MEDN1264	0.548	0.548
**LIPID.P.0351**	MEDN1267	0.566	0.566
**LIPID.P.0351**	MEDN1269	0.816	0.816
**LIPID.P.0351**	MEDN1287	0.318	0.318
**LIPID.P.0351**	MEDP1875	0.443	0.443
**LIPID.P.0351**	LIPID.N.0161	0.381	0.381
**LIPID.P.0351**	LIPID.N.0165	0.619	0.619
**LIPID.P.0351**	LIPID.P.0429	0.340	0.340
**LIPID.P.0351**	LIPID.P.0430	0.479	0.479
**LIPID.P.0351**	LIPID.P.0433	0.261	0.261

**LIPID.P.0351**	LIPID.P.0436	0.371	0.371
**LIPID.P.0351**	LIPID.P.0131	0.793	0.793
**LIPID.P.0351**	MEDP0618	0.880	0.880
**LIPID.P.0351**	MEDP1412	0.782	0.782
**LIPID.P.0351**	MEDP1416	0.525	0.525
**LIPID.P.0351**	MEDP1433	0.944	0.944
**LIPID.P.0351**	MEDP1917	0.736	0.736
**LIPID.P.0351**	LIPID.P.0047	0.640	0.640
**LIPID.P.0351**	LIPID.P.0153	0.010	0.010
**MEDP1875**	McGoon	0.711	0.711
**MEDP1779**	McGoon	0.724	0.724
**MEDP1416**	McGoon	0.726	0.726
**LIPID.P.0352**	LIPID.P.0355	0.988	0.988
**LIPID.P.0352**	LIPID.P.0387	0.256	0.256
**LIPID.P.0352**	LIPID.P.0388	0.253	0.253
**LIPID.P.0352**	LIPID.P.0391	0.167	0.167
**LIPID.P.0352**	MEDN1264	0.528	0.528
**LIPID.P.0352**	MEDN1267	0.518	0.518
**LIPID.P.0352**	MEDN1269	0.782	0.782
**LIPID.P.0352**	MEDN1287	0.276	0.276
**LIPID.P.0352**	MEDP1875	0.401	0.401
**LIPID.P.0352**	LIPID.N.0161	0.354	0.354
**LIPID.P.0352**	LIPID.N.0165	0.599	0.599
**LIPID.P.0352**	LIPID.P.0429	0.334	0.334
**LIPID.P.0352**	LIPID.P.0430	0.479	0.479
**LIPID.P.0352**	LIPID.P.0433	0.259	0.259
**LIPID.P.0352**	LIPID.P.0436	0.356	0.356
**LIPID.P.0352**	LIPID.P.0131	0.845	0.845
**LIPID.P.0352**	MEDP0618	0.835	0.835
**LIPID.P.0352**	MEDP1412	0.698	0.698
**LIPID.P.0352**	MEDP1416	0.406	0.406
**LIPID.P.0352**	MEDP1433	0.916	0.916
**LIPID.P.0352**	MEDP1917	0.688	0.688
**LIPID.P.0352**	LIPID.P.0047	0.572	0.572
**LIPID.P.0352**	LIPID.P.0153	0.087	0.087
**MEDN1287**	McGoon	0.735	0.735
**MEDN1267**	McGoon	0.817	0.817
**LIPID.N.0426**	McGoon	0.842	0.842
**LIPID.P.0355**	LIPID.P.0387	0.257	0.257
**LIPID.P.0355**	LIPID.P.0388	0.252	0.252
**LIPID.P.0355**	LIPID.P.0391	0.173	0.173
**LIPID.P.0355**	MEDN1264	0.546	0.546
**LIPID.P.0355**	MEDN1267	0.521	0.521

**LIPID.P.0355**	MEDN1269	0.772	0.772
**LIPID.P.0355**	MEDN1287	0.267	0.267
**LIPID.P.0355**	MEDP1875	0.433	0.433
**LIPID.P.0355**	LIPID.N.0161	0.368	0.368
**LIPID.P.0355**	LIPID.N.0165	0.604	0.604
**LIPID.P.0355**	LIPID.P.0429	0.357	0.357
**LIPID.P.0355**	LIPID.P.0430	0.498	0.498
**LIPID.P.0355**	LIPID.P.0433	0.297	0.297
**LIPID.P.0355**	LIPID.P.0436	0.372	0.372
**LIPID.P.0355**	LIPID.P.0131	0.860	0.860
**LIPID.P.0355**	MEDP0618	0.808	0.808
**LIPID.P.0355**	MEDP1412	0.695	0.695
**LIPID.P.0355**	MEDP1416	0.395	0.395
**LIPID.P.0355**	MEDP1433	0.893	0.893
**LIPID.P.0355**	MEDP1917	0.666	0.666
**LIPID.P.0355**	LIPID.P.0047	0.570	0.570
**LIPID.P.0355**	LIPID.P.0153	0.196	0.196
**MEDP1697**	McGoon	0.882	0.882
**LIPID.P.0153**	Pre_SPO2	0.181	0.181
**LIPID.N.0229**	Pre_SPO2	0.200	0.200
**LIPID.P.0387**	LIPID.P.0388	0.993	0.993
**LIPID.P.0387**	LIPID.P.0391	0.972	0.972
**LIPID.P.0387**	MEDN1264	0.898	0.898
**LIPID.P.0387**	MEDN1267	0.889	0.889
**LIPID.P.0387**	MEDN1269	0.466	0.466
**LIPID.P.0387**	MEDN1287	0.954	0.954
**LIPID.P.0387**	MEDP1875	0.771	0.771
**LIPID.P.0387**	LIPID.N.0161	0.881	0.881
**LIPID.P.0387**	LIPID.N.0165	0.739	0.739
**LIPID.P.0387**	LIPID.P.0429	0.956	0.956
**LIPID.P.0387**	LIPID.P.0430	0.877	0.877
**LIPID.P.0387**	LIPID.P.0433	0.936	0.936
**LIPID.P.0387**	LIPID.P.0436	0.965	0.965
**LIPID.P.0387**	LIPID.P.0131	0.402	0.402
**LIPID.P.0387**	MEDP1416	0.002	0.002
**LIPID.P.0387**	MEDP1433	0.049	0.049
**LIPID.P.0387**	LIPID.P.0153	0.037	0.037
**LIPID.P.0521**	Pre_SPO2	0.308	0.308
**LIPID.P.0481**	Pre_SPO2	0.319	0.319
**LIPID.P.0506**	Pre_SPO2	0.403	0.403
**MEDP1779**	Pre_SPO2	0.404	0.404
**LIPID.P.0388**	LIPID.P.0391	0.978	0.978
**LIPID.P.0388**	MEDN1264	0.867	0.867

**LIPID.P.0388**	MEDN1267	0.844	0.844
**LIPID.P.0388**	MEDN1269	0.420	0.420
**LIPID.P.0388**	MEDN1287	0.930	0.930
**LIPID.P.0388**	MEDP1875	0.727	0.727
**LIPID.P.0388**	LIPID.N.0161	0.889	0.889
**LIPID.P.0388**	LIPID.N.0165	0.739	0.739
**LIPID.P.0388**	LIPID.P.0429	0.945	0.945
**LIPID.P.0388**	LIPID.P.0430	0.897	0.897
**LIPID.P.0388**	LIPID.P.0433	0.934	0.934
**LIPID.P.0388**	LIPID.P.0436	0.962	0.962
**LIPID.P.0388**	LIPID.P.0131	0.440	0.440
**LIPID.P.0388**	MEDP1433	0.022	0.022
**LIPID.P.0388**	LIPID.P.0153	0.053	0.053
**LIPID.P.0508**	Pre_SPO2	0.409	0.409
**LIPID.P.0759**	Pre_SPO2	0.414	0.414
**LIPID.N.0276**	Pre_SPO2	0.420	0.420
**LIPID.P.0491**	Pre_SPO2	0.422	0.422
**LIPID.P.0391**	MEDN1264	0.803	0.803
**LIPID.P.0391**	MEDN1267	0.796	0.796
**LIPID.P.0391**	MEDN1269	0.339	0.339
**LIPID.P.0391**	MEDN1287	0.899	0.899
**LIPID.P.0391**	MEDP1875	0.659	0.659
**LIPID.P.0391**	LIPID.N.0161	0.925	0.925
**LIPID.P.0391**	LIPID.N.0165	0.763	0.763
**LIPID.P.0391**	LIPID.P.0429	0.885	0.885
**LIPID.P.0391**	LIPID.P.0430	0.869	0.869
**LIPID.P.0391**	LIPID.P.0433	0.912	0.912
**LIPID.P.0391**	LIPID.P.0436	0.950	0.950
**LIPID.P.0391**	LIPID.P.0131	0.351	0.351
**LIPID.P.0391**	LIPID.P.0153	0.006	0.006
**LIPID.N.0250**	Pre_SPO2	0.437	0.437
**MEDP0618**	Pre_SPO2	0.444	0.444
**MEDP1699**	Pre_SPO2	0.456	0.456
**MEDP1917**	Pre_SPO2	0.464	0.464
**MEDN1264**	MEDN1267	0.977	0.977
**MEDN1264**	MEDN1269	0.736	0.736
**MEDN1264**	MEDN1287	0.887	0.887
**MEDN1264**	MEDP1875	0.873	0.873
**MEDN1264**	LIPID.N.0161	0.768	0.768
**MEDN1264**	LIPID.N.0165	0.749	0.749
**MEDN1264**	LIPID.P.0429	0.948	0.948
**MEDN1264**	LIPID.P.0430	0.821	0.821
**MEDN1264**	LIPID.P.0433	0.874	0.874

**MEDN1264**	LIPID.P.0436	0.895	0.895
**MEDN1264**	LIPID.P.0131	0.564	0.564
**MEDN1264**	MEDP0618	0.299	0.299
**MEDN1264**	MEDP1412	0.238	0.238
**MEDN1264**	MEDP1416	0.148	0.148
**MEDN1264**	MEDP1433	0.350	0.350
**MEDN1264**	MEDP1917	0.219	0.219
**MEDN1264**	LIPID.P.0047	0.215	0.215
**MEDN1264**	LIPID.P.0153	0.154	0.154
**LIPID.N.0119**	Pre_SPO2	0.481	0.481
**LIPID.N.0132**	Pre_SPO2	0.503	0.503
**LIPID.N.0413**	Pre_SPO2	0.518	0.518
**MEDN1267**	MEDN1269	0.762	0.762
**MEDN1267**	MEDN1287	0.900	0.900
**MEDN1267**	MEDP1875	0.862	0.862
**MEDN1267**	LIPID.N.0161	0.762	0.762
**MEDN1267**	LIPID.N.0165	0.753	0.753
**MEDN1267**	LIPID.P.0429	0.898	0.898
**MEDN1267**	LIPID.P.0430	0.773	0.773
**MEDN1267**	LIPID.P.0433	0.818	0.818
**MEDN1267**	LIPID.P.0436	0.869	0.869
**MEDN1267**	LIPID.P.0131	0.469	0.469
**MEDN1267**	MEDP0618	0.366	0.366
**MEDN1267**	MEDP1412	0.336	0.336
**MEDN1267**	MEDP1416	0.293	0.293
**MEDN1267**	MEDP1433	0.398	0.398
**MEDN1267**	MEDP1917	0.286	0.286
**MEDN1267**	LIPID.P.0047	0.297	0.297
**MEDP1416**	Pre_SPO2	0.521	0.521
**LIPID.P.0047**	Pre_SPO2	0.522	0.522
**MEDP1433**	Pre_SPO2	0.523	0.523
**MEDN1269**	MEDN1287	0.448	0.448
**MEDN1269**	MEDP1875	0.551	0.551
**MEDN1269**	LIPID.N.0161	0.424	0.424
**MEDN1269**	LIPID.N.0165	0.683	0.683
**MEDN1269**	LIPID.P.0429	0.532	0.532
**MEDN1269**	LIPID.P.0430	0.451	0.451
**MEDN1269**	LIPID.P.0433	0.346	0.346
**MEDN1269**	LIPID.P.0436	0.444	0.444
**MEDN1269**	LIPID.P.0131	0.615	0.615
**MEDN1269**	MEDP0618	0.729	0.729
**MEDN1269**	MEDP1412	0.632	0.632
**MEDN1269**	MEDP1416	0.431	0.431

**MEDN1269**	MEDP1433	0.804	0.804
**MEDN1269**	MEDP1917	0.714	0.714
**MEDN1269**	LIPID.P.0047	0.667	0.667
**LIPID.P.0391**	Pre_SPO2	0.528	0.528
**LIPID.P.0352**	Pre_SPO2	0.532	0.532
**LIPID.P.0388**	Pre_SPO2	0.547	0.547
**MEDN1287**	MEDP1875	0.875	0.875
**MEDN1287**	LIPID.N.0161	0.834	0.834
**MEDN1287**	LIPID.N.0165	0.648	0.648
**MEDN1287**	LIPID.P.0429	0.926	0.926
**MEDN1287**	LIPID.P.0430	0.855	0.855
**MEDN1287**	LIPID.P.0433	0.915	0.915
**MEDN1287**	LIPID.P.0436	0.932	0.932
**MEDN1287**	LIPID.P.0131	0.360	0.360
**MEDN1287**	MEDP0618	0.090	0.090
**MEDN1287**	MEDP1412	0.105	0.105
**MEDN1287**	MEDP1416	0.183	0.183
**MEDN1287**	MEDP1433	0.099	0.099
**MEDN1287**	MEDP1917	0.015	0.015
**MEDN1287**	LIPID.P.0047	0.046	0.046
**MEDN1287**	LIPID.P.0153	0.067	0.067
**MEDP1412**	Pre_SPO2	0.564	0.564
**LIPID.P.0387**	Pre_SPO2	0.573	0.573
**LIPID.P.0351**	Pre_SPO2	0.586	0.586
**LIPID.P.0355**	Pre_SPO2	0.589	0.589
**MEDP1875**	LIPID.N.0161	0.658	0.658
**MEDP1875**	LIPID.N.0165	0.503	0.503
**MEDP1875**	LIPID.P.0429	0.856	0.856
**MEDP1875**	LIPID.P.0430	0.772	0.772
**MEDP1875**	LIPID.P.0433	0.813	0.813
**MEDP1875**	LIPID.P.0436	0.767	0.767
**MEDP1875**	LIPID.P.0131	0.457	0.457
**MEDP1875**	MEDP0618	0.241	0.241
**MEDP1875**	MEDP1412	0.347	0.347
**MEDP1875**	MEDP1416	0.371	0.371
**MEDP1875**	MEDP1433	0.261	0.261
**MEDP1875**	MEDP1917	0.179	0.179
**MEDP1875**	LIPID.P.0047	0.241	0.241
**MEDP1875**	LIPID.P.0550	0.016	0.016
**MEDP1875**	LIPID.P.0153	0.309	0.309
**MEDN1287**	Pre_SPO2	0.589	0.589
**LIPID.P.0433**	Pre_SPO2	0.603	0.603
**LIPID.N.0161**	LIPID.N.0165	0.895	0.895

**LIPID.N.0161**	LIPID.P.0429	0.809	0.809
**LIPID.N.0161**	LIPID.P.0430	0.934	0.934
**LIPID.N.0161**	LIPID.P.0433	0.860	0.860
**LIPID.N.0161**	LIPID.P.0436	0.914	0.914
**LIPID.N.0161**	LIPID.P.0131	0.535	0.535
**LIPID.N.0161**	MEDP0618	0.116	0.116
**LIPID.N.0161**	MEDP1412	0.101	0.101
**LIPID.N.0161**	MEDP1416	0.151	0.151
**LIPID.N.0161**	MEDP1433	0.183	0.183
**LIPID.N.0161**	MEDP1917	0.096	0.096
**LIPID.N.0161**	LIPID.P.0047	0.095	0.095
**LIPID.N.0161**	LIPID.P.0153	0.043	0.043
**LIPID.N.0426**	Pre_SPO2	0.605	0.605
**MEDP1697**	Pre_SPO2	0.608	0.608
**LIPID.P.0436**	Pre_SPO2	0.608	0.608
**LIPID.P.0480**	Pre_SPO2	0.629	0.629
**LIPID.N.0165**	LIPID.P.0429	0.684	0.684
**LIPID.N.0165**	LIPID.P.0430	0.804	0.804
**LIPID.N.0165**	LIPID.P.0433	0.671	0.671
**LIPID.N.0165**	LIPID.P.0436	0.783	0.783
**LIPID.N.0165**	LIPID.P.0131	0.655	0.655
**LIPID.N.0165**	MEDP0618	0.408	0.408
**LIPID.N.0165**	MEDP1412	0.291	0.291
**LIPID.N.0165**	MEDP1416	0.214	0.214
**LIPID.N.0165**	MEDP1433	0.494	0.494
**LIPID.N.0165**	MEDP1917	0.406	0.406
**LIPID.N.0165**	LIPID.P.0047	0.352	0.352
**LIPID.P.0429**	Pre_SPO2	0.646	0.646
**LIPID.P.0131**	Pre_SPO2	0.648	0.648
**MEDN1269**	Pre_SPO2	0.662	0.662
**LIPID.P.0429**	LIPID.P.0430	0.879	0.879
**LIPID.P.0429**	LIPID.P.0433	0.956	0.956
**LIPID.P.0429**	LIPID.P.0436	0.942	0.942
**LIPID.P.0429**	LIPID.P.0131	0.513	0.513
**LIPID.P.0429**	MEDP0618	0.053	0.053
**LIPID.P.0429**	MEDP1412	0.015	0.015
**LIPID.P.0429**	MEDP1433	0.110	0.110
**LIPID.P.0429**	MEDP1917	0.000	0.000
**LIPID.P.0429**	LIPID.P.0047	0.036	0.036
**LIPID.P.0429**	LIPID.P.0628	0.030	0.030
**LIPID.P.0429**	LIPID.P.0153	0.248	0.248
**MEDP1686**	Pre_SPO2	0.668	0.668
**MEDN1267**	Pre_SPO2	0.685	0.685

**MEDN1264**	Pre_SPO2	0.693	0.693
**LIPID.P.0430**	LIPID.P.0433	0.906	0.906
**LIPID.P.0430**	LIPID.P.0436	0.920	0.920
**LIPID.P.0430**	LIPID.P.0131	0.708	0.708
**LIPID.P.0430**	MEDP0618	0.142	0.142
**LIPID.P.0430**	MEDP1412	0.138	0.138
**LIPID.P.0430**	MEDP1416	0.113	0.113
**LIPID.P.0430**	MEDP1433	0.241	0.241
**LIPID.P.0430**	MEDP1917	0.066	0.066
**LIPID.P.0430**	LIPID.P.0047	0.073	0.073
**LIPID.P.0430**	LIPID.P.0153	0.251	0.251
**LIPID.N.0161**	Pre_SPO2	0.722	0.722
**LIPID.N.0165**	Pre_SPO2	0.729	0.729
**MEDP1875**	Pre_SPO2	0.741	0.741
**LIPID.P.0433**	LIPID.P.0436	0.973	0.973
**LIPID.P.0433**	LIPID.P.0131	0.467	0.467
**LIPID.P.0433**	LIPID.P.0550	0.004	0.004
**LIPID.P.0433**	LIPID.P.0153	0.368	0.368
**LIPID.P.0430**	Pre_SPO2	0.747	0.747
**LIPID.P.0153**	RVAW	0.108	0.108
**LIPID.P.0628**	RVAW	0.391	0.391
**LIPID.P.0436**	LIPID.P.0131	0.497	0.497
**LIPID.P.0436**	MEDP0618	0.089	0.089
**LIPID.P.0436**	MEDP1412	0.029	0.029
**LIPID.P.0436**	MEDP1416	0.013	0.013
**LIPID.P.0436**	MEDP1433	0.120	0.120
**LIPID.P.0436**	LIPID.P.0153	0.194	0.194
**LIPID.P.0363**	RVAW	0.477	0.477
**LIPID.P.0550**	RVAW	0.520	0.520
**LIPID.P.0495**	RVAW	0.560	0.560
**RVOTd**	RVAW	0.705	0.705
**LIPID.P.0131**	MEDP0618	0.512	0.512
**LIPID.P.0131**	MEDP1412	0.386	0.386
**LIPID.P.0131**	MEDP1416	0.109	0.109
**LIPID.P.0131**	MEDP1433	0.651	0.651
**LIPID.P.0131**	MEDP1917	0.427	0.427
**LIPID.P.0131**	LIPID.P.0047	0.335	0.335
**LIPID.P.0131**	LIPID.P.0550	0.044	0.044
**LIPID.P.0131**	LIPID.P.0628	0.086	0.086
**LIPID.P.0131**	LIPID.P.0153	0.321	0.321
**LIPID.N.0119**	RVOT	0.002	0.002
**LIPID.P.0047**	RVOT	0.033	0.033
**MEDN1287**	RVOT	0.052	0.052

**MEDP0618**	MEDP1412	0.893	0.893
**MEDP0618**	MEDP1416	0.671	0.671
**MEDP0618**	MEDP1433	0.953	0.953
**MEDP0618**	MEDP1917	0.898	0.898
**MEDP0618**	LIPID.P.0047	0.785	0.785
**LIPID.P.0433**	RVOT	0.061	0.061
**MEDP1699**	RVOT	0.070	0.070
**LIPID.P.0481**	RVOT	0.078	0.078
**MEDP1412**	MEDP1416	0.895	0.895
**MEDP1412**	MEDP1433	0.876	0.876
**MEDP1412**	MEDP1917	0.797	0.797
**MEDP1412**	LIPID.P.0047	0.797	0.797
**MEDP1917**	RVOT	0.083	0.083
**LIPID.P.0430**	RVOT	0.085	0.085
**LIPID.P.0131**	RVOT	0.085	0.085
**MEDP1416**	MEDP1433	0.632	0.632
**MEDP1416**	MEDP1917	0.660	0.660
**MEDP1416**	LIPID.P.0047	0.728	0.728
**Pre_SPO2**	RVOT	0.096	0.096
**MEDP1697**	RVOT	0.102	0.102
**LIPID.P.0429**	RVOT	0.102	0.102
**MEDP1433**	MEDP1917	0.855	0.855
**MEDP1433**	LIPID.P.0047	0.761	0.761
**MEDP1412**	RVOT	0.106	0.106
**MEDP1779**	RVOT	0.110	0.110
**LIPID.N.0250**	RVOT	0.128	0.128
**MEDP1917**	LIPID.P.0047	0.941	0.941
**LIPID.N.0413**	RVOT	0.135	0.135
**LIPID.N.0132**	RVOT	0.162	0.162
**MEDP1686**	RVOT	0.170	0.170
**MEDP0618**	RVOT	0.198	0.198
**LIPID.P.0480**	RVOT	0.212	0.212
**LIPID.P.0436**	RVOT	0.217	0.217
**LIPID.P.0495**	LIPID.P.0550	0.831	0.831
**LIPID.P.0495**	LIPID.P.0628	0.474	0.474
**LIPID.P.0495**	LIPID.P.0363	0.966	0.966
**LIPID.P.0495**	LIPID.P.0153	0.235	0.235
**LIPID.P.0355**	RVOT	0.225	0.225
**MEDN1264**	RVOT	0.227	0.227
**LIPID.P.0352**	RVOT	0.233	0.233
**LIPID.P.0550**	LIPID.P.0628	0.507	0.507
**LIPID.P.0550**	LIPID.P.0363	0.841	0.841
**LIPID.P.0550**	LIPID.P.0153	0.678	0.678

**LIPID.P.0387**	RVOT	0.236	0.236
**LIPID.P.0388**	RVOT	0.236	0.236
**LIPID.N.0161**	RVOT	0.256	0.256
**LIPID.P.0628**	LIPID.P.0363	0.438	0.438
**LIPID.P.0628**	LIPID.P.0153	0.320	0.320
**LIPID.P.0491**	RVOT	0.266	0.266
**MEDP1433**	RVOT	0.268	0.268
**LIPID.P.0506**	RVOT	0.268	0.268
**LIPID.P.0363**	LIPID.P.0153	0.250	0.250
**LIPID.P.0351**	RVOT	0.273	0.273
**MEDN1267**	RVOT	0.284	0.284
**LIPID.N.0426**	RVOT	0.290	0.290
**LIPID.P.0391**	RVOT	0.294	0.294
**LIPID.P.0508**	RVOT	0.325	0.325
**LIPID.P.0521**	RVOT	0.334	0.334
**LIPID.P.0759**	RVOT	0.350	0.350
**MEDN1269**	RVOT	0.392	0.392
**LIPID.N.0165**	RVOT	0.470	0.470
**LIPID.P.0628**	RVOTd	0.126	0.126
**LIPID.P.0495**	RVOTd	0.246	0.246
**LIPID.P.0363**	RVOTd	0.249	0.249
**LIPID.P.0153**	RVOTd	0.398	0.398
**LIPID.P.0550**	RVOTd	0.478	0.478
