# Supplementary material for: Circular Network of Coregulated Sphingolipids Dictates Chronic Hypoxia Damage in Patients With Tetralogy of Fallot
Source: Front Cardiovasc Med. 2022 Jan 13;8:780123. doi: 10.3389/fcvm.2021.780123 (PMC8792512; doi:10.3389/fcvm.2021.780123)
Supplement: Supplementary Table 6 — Partial correlation between sphingomyelin-related metabolites and clinical phenotypes in patients with TOF in right atrial biopsies. [file Table_6.pdf]

Table S6. The partial correlation between sphingomyelin related metabolites and clinical phenotypes in patients with TOF in right atrial biopsies.

source	target	pearson_corr	weight
**LIPID.N.0465**	LIPID.P.0689	0.526	0.526
**LIPID.N.0465**	LIPID.N.0397	0.722	0.722
**LIPID.N.0465**	LIPID.N.0429	0.768	0.768
**LIPID.N.0465**	LIPID.P.0398	0.524	0.524
**LIPID.N.0465**	LIPID.N.0188	0.493	0.493
**LIPID.N.0465**	LIPID.N.0196	0.598	0.598
**LIPID.N.0465**	LIPID.N.0151	0.706	0.706
**LIPID.N.0465**	LIPID.N.0152	0.685	0.685
**LIPID.P.0048**	EF	0.085	0.085
**LIPID.N.0188**	EF	0.101	0.101
**LIPID.P.0039**	EF	0.143	0.143
**LIPID.N.0465**	EF	0.200	0.200
**LIPID.P.0689**	LIPID.N.0397	0.476	0.476
**LIPID.P.0689**	LIPID.N.0429	0.302	0.302
**LIPID.P.0689**	LIPID.P.0398	0.663	0.663
**LIPID.P.0689**	LIPID.N.0188	0.927	0.927
**LIPID.P.0689**	LIPID.N.0196	0.699	0.699
**LIPID.P.0689**	LIPID.N.0151	0.792	0.792
**LIPID.P.0689**	LIPID.N.0152	0.797	0.797
**LIPID.P.0013**	EF	0.264	0.264
**LIPID.P.0555**	EF	0.362	0.362
**LIPID.P.0037**	EF	0.374	0.374
**LIPID.N.0397**	LIPID.N.0429	0.523	0.523
**LIPID.N.0397**	LIPID.P.0398	0.746	0.746
**LIPID.N.0397**	LIPID.N.0188	0.430	0.430
**LIPID.N.0397**	LIPID.N.0196	0.521	0.521
**LIPID.N.0397**	LIPID.N.0151	0.613	0.613
**LIPID.N.0397**	LIPID.N.0152	0.681	0.681
**LIPID.N.0397**	LIPID.P.0505	0.072	0.072
**LIPID.N.0397**	LIPID.P.0508	0.179	0.179
**LIPID.N.0397**	LIPID.P.0520	0.136	0.136
**LIPID.N.0397**	LIPID.P.0111	0.165	0.165
**LIPID.P.0047**	EF	0.481	0.481
**LIPID.P.0035**	EF	0.511	0.511
**LIPID.N.0188**	IVS	0.459	0.459
**LIPID.N.0429**	LIPID.P.0398	0.123	0.123
**LIPID.N.0429**	LIPID.N.0188	0.129	0.129
**LIPID.N.0429**	LIPID.N.0196	0.356	0.356

**LIPID.N.0429**	LIPID.N.0151	0.447	0.447
**LIPID.N.0429**	LIPID.N.0152	0.474	0.474
**LIPID.P.0689**	IVS	0.490	0.490
**LIPID.P.0398**	IVS	0.501	0.501
**LIPID.N.0429**	IVS	0.549	0.549
**LIPID.N.0196**	IVS	0.556	0.556
**LIPID.P.0398**	LIPID.N.0188	0.685	0.685
**LIPID.P.0398**	LIPID.N.0196	0.678	0.678
**LIPID.P.0398**	LIPID.N.0151	0.681	0.681
**LIPID.P.0398**	LIPID.N.0152	0.744	0.744
**LIPID.P.0398**	LIPID.P.0505	0.244	0.244
**LIPID.P.0398**	LIPID.P.0508	0.313	0.313
**LIPID.P.0398**	LIPID.P.0520	0.319	0.319
**LIPID.P.0398**	LIPID.P.0567	0.120	0.120
**LIPID.P.0398**	LIPID.P.0111	0.350	0.350
**LIPID.P.0398**	LIPID.P.0039	0.021	0.021
**LIPID.N.0465**	IVS	0.636	0.636
**LIPID.N.0152**	IVS	0.641	0.641
**LIPID.N.0151**	IVS	0.651	0.651
**LIPID.N.0188**	LIPID.N.0196	0.702	0.702
**LIPID.N.0188**	LIPID.N.0151	0.800	0.800
**LIPID.N.0188**	LIPID.N.0152	0.779	0.779
**RVOTd**	IVS	0.678	0.678
**LIPID.N.0397**	IVS	0.737	0.737
**RVAW**	IVS	0.877	0.877
**EF**	McGoon	0.133	0.133
**LIPID.N.0196**	LIPID.N.0151	0.934	0.934
**LIPID.N.0196**	LIPID.N.0152	0.931	0.931
**LIPID.N.0196**	LIPID.P.0007	0.015	0.015
**LIPID.P.0007**	McGoon	0.187	0.187
**LIPID.P.0010**	McGoon	0.194	0.194
**LIPID.P.0508**	McGoon	0.247	0.247
**LIPID.N.0151**	LIPID.N.0152	0.976	0.976
**RVOT**	McGoon	0.258	0.258
**LIPID.P.0111**	McGoon	0.305	0.305
**LIPID.P.0520**	McGoon	0.307	0.307
**LIPID.P.0505**	McGoon	0.350	0.350
**LIPID.P.0013**	McGoon	0.428	0.428
**LIPID.P.0567**	McGoon	0.461	0.461
**LIPID.P.0505**	LIPID.P.0508	0.870	0.870
**LIPID.P.0505**	LIPID.P.0520	0.980	0.980
**LIPID.P.0505**	LIPID.P.0555	0.583	0.583
**LIPID.P.0505**	LIPID.P.0567	0.873	0.873

**LIPID.P.0505**	LIPID.P.0111	0.947	0.947
**LIPID.P.0505**	LIPID.P.0010	0.029	0.029
**LIPID.P.0505**	LIPID.P.0013	0.301	0.301
**LIPID.P.0505**	LIPID.P.0035	0.480	0.480
**LIPID.P.0505**	LIPID.P.0037	0.594	0.594
**LIPID.P.0505**	LIPID.P.0039	0.811	0.811
**LIPID.P.0505**	LIPID.P.0047	0.429	0.429
**LIPID.P.0505**	LIPID.P.0048	0.820	0.820
**LIPID.P.0035**	McGoon	0.479	0.479
**LIPID.P.0048**	McGoon	0.511	0.511
**LIPID.P.0508**	LIPID.P.0520	0.931	0.931
**LIPID.P.0508**	LIPID.P.0555	0.385	0.385
**LIPID.P.0508**	LIPID.P.0567	0.891	0.891
**LIPID.P.0508**	LIPID.P.0111	0.945	0.945
**LIPID.P.0508**	LIPID.P.0007	0.230	0.230
**LIPID.P.0508**	LIPID.P.0010	0.321	0.321
**LIPID.P.0508**	LIPID.P.0013	0.339	0.339
**LIPID.P.0508**	LIPID.P.0035	0.351	0.351
**LIPID.P.0508**	LIPID.P.0037	0.396	0.396
**LIPID.P.0508**	LIPID.P.0039	0.677	0.677
**LIPID.P.0508**	LIPID.P.0047	0.260	0.260
**LIPID.P.0508**	LIPID.P.0048	0.695	0.695
**LIPID.P.0039**	McGoon	0.514	0.514
**LIPID.P.0555**	McGoon	0.570	0.570
**LIPID.P.0047**	McGoon	0.629	0.629
**LIPID.P.0520**	LIPID.P.0555	0.467	0.467
**LIPID.P.0520**	LIPID.P.0567	0.887	0.887
**LIPID.P.0520**	LIPID.P.0111	0.956	0.956
**LIPID.P.0520**	LIPID.P.0007	0.074	0.074
**LIPID.P.0520**	LIPID.P.0010	0.161	0.161
**LIPID.P.0520**	LIPID.P.0013	0.292	0.292
**LIPID.P.0520**	LIPID.P.0035	0.381	0.381
**LIPID.P.0520**	LIPID.P.0037	0.483	0.483
**LIPID.P.0520**	LIPID.P.0039	0.734	0.734
**LIPID.P.0520**	LIPID.P.0047	0.316	0.316
**LIPID.P.0520**	LIPID.P.0048	0.748	0.748
**Pre_SPO2**	McGoon	0.654	0.654
**LIPID.P.0037**	McGoon	0.658	0.658
**LIPID.P.0555**	LIPID.P.0567	0.416	0.416
**LIPID.P.0555**	LIPID.P.0111	0.547	0.547
**LIPID.P.0555**	LIPID.P.0013	0.550	0.550
**LIPID.P.0555**	LIPID.P.0035	0.928	0.928
**LIPID.P.0555**	LIPID.P.0037	0.975	0.975

**LIPID.P.0555**	LIPID.P.0039	0.925	0.925
**LIPID.P.0555**	LIPID.P.0047	0.916	0.916
**LIPID.P.0555**	LIPID.P.0048	0.903	0.903
**LIPID.P.0007**	Pre_SPO2	0.178	0.178
**LIPID.P.0010**	Pre_SPO2	0.240	0.240
**LIPID.P.0508**	Pre_SPO2	0.289	0.289
**LIPID.P.0567**	LIPID.P.0111	0.884	0.884
**LIPID.P.0567**	LIPID.P.0007	0.109	0.109
**LIPID.P.0567**	LIPID.P.0010	0.210	0.210
**LIPID.P.0567**	LIPID.P.0013	0.282	0.282
**LIPID.P.0567**	LIPID.P.0035	0.334	0.334
**LIPID.P.0567**	LIPID.P.0037	0.452	0.452
**LIPID.P.0567**	LIPID.P.0039	0.692	0.692
**LIPID.P.0567**	LIPID.P.0047	0.293	0.293
**LIPID.P.0567**	LIPID.P.0048	0.715	0.715
**LIPID.P.0111**	Pre_SPO2	0.325	0.325
**LIPID.P.0035**	Pre_SPO2	0.328	0.328
**LIPID.P.0013**	Pre_SPO2	0.404	0.404
**LIPID.P.0111**	LIPID.P.0007	0.003	0.003
**LIPID.P.0111**	LIPID.P.0010	0.092	0.092
**LIPID.P.0111**	LIPID.P.0013	0.317	0.317
**LIPID.P.0111**	LIPID.P.0035	0.488	0.488
**LIPID.P.0111**	LIPID.P.0037	0.538	0.538
**LIPID.P.0111**	LIPID.P.0039	0.808	0.808
**LIPID.P.0111**	LIPID.P.0047	0.370	0.370
**LIPID.P.0111**	LIPID.P.0048	0.790	0.790
**LIPID.P.0520**	Pre_SPO2	0.408	0.408
**LIPID.P.0555**	Pre_SPO2	0.427	0.427
**LIPID.P.0007**	LIPID.P.0010	0.990	0.990
**LIPID.P.0007**	LIPID.P.0013	0.544	0.544
**LIPID.P.0007**	LIPID.P.0035	0.001	0.001
**LIPID.P.0039**	Pre_SPO2	0.440	0.440
**LIPID.P.0047**	Pre_SPO2	0.470	0.470
**LIPID.P.0505**	Pre_SPO2	0.474	0.474
**LIPID.P.0010**	LIPID.P.0013	0.597	0.597
**LIPID.P.0010**	LIPID.P.0035	0.054	0.054
**LIPID.P.0010**	LIPID.P.0048	0.046	0.046
**LIPID.P.0037**	Pre_SPO2	0.488	0.488
**LIPID.P.0048**	Pre_SPO2	0.489	0.489
**LIPID.P.0567**	Pre_SPO2	0.520	0.520
**LIPID.P.0013**	LIPID.P.0035	0.759	0.759
**LIPID.P.0013**	LIPID.P.0037	0.621	0.621
**LIPID.P.0013**	LIPID.P.0039	0.534	0.534

**LIPID.P.0013**	LIPID.P.0047	0.688	0.688
**LIPID.P.0013**	LIPID.P.0048	0.578	0.578
**LIPID.P.0398**	RVAW	0.400	0.400
**LIPID.N.0196**	RVAW	0.462	0.462
**LIPID.N.0188**	RVAW	0.582	0.582
**LIPID.N.0152**	RVAW	0.585	0.585
**LIPID.P.0035**	LIPID.P.0037	0.923	0.923
**LIPID.P.0035**	LIPID.P.0039	0.852	0.852
**LIPID.P.0035**	LIPID.P.0047	0.920	0.920
**LIPID.P.0035**	LIPID.P.0048	0.831	0.831
**LIPID.P.0689**	RVAW	0.593	0.593
**LIPID.N.0429**	RVAW	0.616	0.616
**LIPID.N.0151**	RVAW	0.648	0.648
**LIPID.P.0037**	LIPID.P.0039	0.906	0.906
**LIPID.P.0037**	LIPID.P.0047	0.964	0.964
**LIPID.P.0037**	LIPID.P.0048	0.906	0.906
**LIPID.N.0397**	RVAW	0.661	0.661
**RVOTd**	RVAW	0.705	0.705
**LIPID.N.0465**	RVAW	0.742	0.742
**LIPID.P.0037**	RVOT	0.042	0.042
**LIPID.P.0039**	LIPID.P.0047	0.784	0.784
**LIPID.P.0039**	LIPID.P.0048	0.982	0.982
**LIPID.P.0567**	RVOT	0.042	0.042
**LIPID.P.0508**	RVOT	0.044	0.044
**LIPID.P.0048**	RVOT	0.055	0.055
**LIPID.P.0047**	LIPID.P.0048	0.800	0.800
**LIPID.P.0047**	RVOT	0.092	0.092
**Pre_SPO2**	RVOT	0.096	0.096
**LIPID.N.0429**	RVOT	0.215	0.215
**LIPID.P.0013**	RVOT	0.354	0.354
**LIPID.P.0007**	RVOT	0.484	0.484
**LIPID.P.0010**	RVOT	0.500	0.500
**LIPID.P.0398**	RVOTd	0.311	0.311
**LIPID.N.0397**	RVOTd	0.366	0.366
**LIPID.N.0196**	RVOTd	0.487	0.487
**LIPID.N.0465**	RVOTd	0.499	0.499
**LIPID.N.0188**	RVOTd	0.542	0.542
**LIPID.N.0151**	RVOTd	0.594	0.594
**LIPID.N.0152**	RVOTd	0.609	0.609
**LIPID.N.0429**	RVOTd	0.682	0.682
**LIPID.P.0689**	RVOTd	0.695	0.695
